# Beyond prevalence: A complete catchment eye health system profile using rapid assessment of avoidable blindness, a population-based eye health survey, and the first implementation of the newly developed health system module

**DOI:** 10.1371/journal.pone.0354744

**Published:** 2026-08-10

**Authors:** Shalinder Sabherwal, Ian McCormick, Mohd Javed, Jacqueline Ramke, Shamanna B. R, Sandeep Buttan, Atanu Majumdar, Pushkar Silwal, Simrat Chandi, Basitali Lakhani, Shreya Tyagi, Utsav Deep, Vaibhav Jain, Andrew Bastawrous

**Affiliations:** 1 Dr Shroff’s Charity Eye Hospital, Kedarnath Lane, Daryaganj, New Delhi, India; 2 London School of Hygiene & Tropical Medicine, London, United Kingdom; 3 University of Hyderabad, C.R. Rao Road, Central University Gachibowli, Hyderabad, Telangana, India; 4 University of Auckland, Auckland Central, Auckland, New Zealand; LV Prasad Eye Institute, INDIA

## Abstract

**Background:**

Planning eye care services often relies on either population need or facility capability, but seldom both. We provide a complete catchment-level assessment by integrating Rapid Assessment of Avoidable Blindness epidemiological estimates with the new Health System Module..

**Methods:**

A population-based survey using the Rapid Assessment of Avoidable Blindness, including a diabetic retinopathy module, was conducted in a defined service area of a surgical centre and its affiliated vision centres. The Health System Module captured cataract, refractive, and diabetic retinopathy service availability and data quality across all major providers.

**Results:**

Of the 4,095 participants enumerated, aged 50 and older, 3,867 (94.4%) were examined. The age- and sex-adjusted prevalence of blindness was 2.2%, higher in women (2.8%) than men (1.6%) (p < 0.001), while visual impairment affected 29.5%. Unoperated cataract accounted for 76.5% of blindness, 84.3% of severe, and 54.7% of moderate impairment; uncorrected refractive error caused 79.2% of mild impairment. Cataract surgical coverage was 59.6% at 6/12 and 70.3% at 6/18, with a 36.6% quality gap. A higher proportion of operated patients had a good outcome (74.4%) when surgery was performed within 2 years, compared with 57.0% when performed earlier (p < 0.0001). Distance refractive error coverage was 14.0%, lower among women (9.5%) than men (19.5%) (p < 0.001). Of the 14 providers, data on cataract surgeries were available from 12, on glasses from four, on visual acuity before and after surgery from one, and on DR services from none. Combined RAAB and Health System Module results showed that although the cataract surgical rate of 7,165 surgeries per million people annually exceeded the national average, an estimated 41,165 individuals had blindness or visual impairment from cataract in both eyes.

**Conclusion:**

Integrating RAAB with a structured health system assessment enabled a unique, comprehensive situational analysis linking epidemiological needs with service capacity. It also highlighted data gaps, feasibility and operational limitations regarding data for cataract, refractive, and diabetic retinopathy services in the region. This model can guide regional planning and strengthen monitoring of effective coverage. However, challenges in data collection can reduce the programmatic utility of the Health System Module.

## Introduction

There is a high magnitude of blindness and vision impairment in low-and middle-income countries, including India, [1] highlighting the need for effective planning of eye care services. Shroff’s Charity Eye Hospital operates a network of eye care facilities in northern India using a pyramidal approach to eye care. [2] The organization provides services in Uttar Pradesh, India’s most populous state in the north, [3] through five secondary surgical centres in five districts, as well as more than 100 connected primary eye care centres (vision centres, [VCs]). These centres deliver primary eye care and refer patients to the secondary-level surgical centres.

A national Rapid Assessment of Avoidable Blindness (RAAB) survey was conducted in India between 2015 and 2019, revealing variations in eye health outcomes by region, education, and age. [4] The highest prevalence of blindness was observed in one district of Uttar Pradesh, but there was variation among the three districts selected from the state. One of the hospitals in our network is located in Lakhimpur Kheri district in Uttar Pradesh, which was not sampled as part of the national survey and lacked population eye health data. At the time of our study, this hospital was the primary service provider in its catchment area, with a network of seven VCs serving a population of approximately 2 million people. A plan was in place to expand services in the area, so to assess the need for eye care services in the population, we conducted a RAAB survey. [5] in its latest digital format, RAAB7. [6]

Although Dr Shroff’s Charity Eye Hospital was the leading service provider, other eye care providers operated in the same area. Therefore, we aimed to understand our service delivery in the context of both the population’s needs and the services provided by other eye care organizations. To do this, we implemented the optional RAAB Health System module. The RAAB Health System Module, recently developed by the RAAB Steering Group members, allows for the collection of structured, facility-level data on the availability, capacity, and quality of services required to fulfil the needs identified in RAAB surveys. The Health System Module is an innovation that enhances RAAB by rapidly identifying gaps in current eye care services. This module collects information on policies related to eye services. It captures services offered by both government and non-government eye care providers for three common causes of vision loss: cataract, uncorrected refractive error, and diabetic retinopathy (DR). Since RAABs are typically conducted at the subnational level, this module provides a quick, user-friendly tool to support eye care planning within a survey area. While RAAB results can help estimate the burden of eye diseases, data on available services are also needed to plan at the district level. This module enables a relatively quick assessment of service gaps for cataract, refractive error, and DR services. The data to be collected in the module include the outputs and outcomes of cataract surgeries, the number of glasses dispensed, vision with glasses, the number of individuals screened and treated for DR, and the human resources at the respective centres providing services. This can support the planning needed to scale up services, improve service quality, and plan the workforce.

In this study, we present a model for a rapid situational analysis within the catchment area of a service provider, utilising results from a RAAB survey and the Health System module.

## Materials and methods

### Study design, setting and period

**Fig 1:**
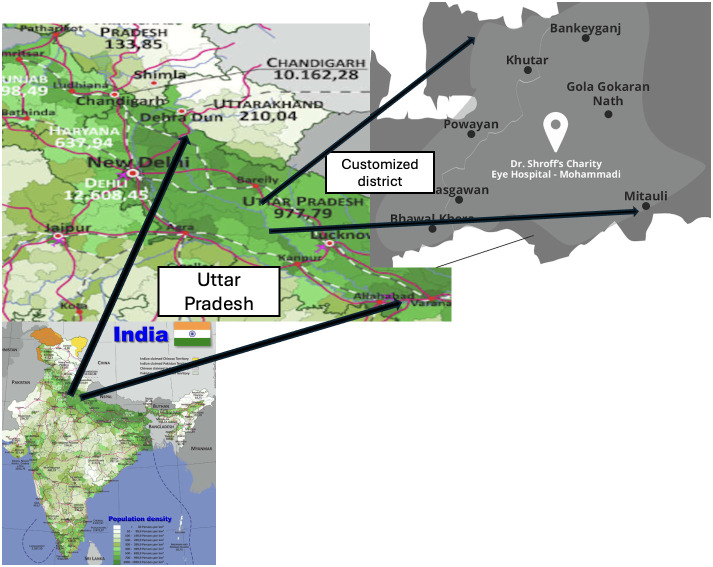
A geographical map illustrating the area where the study was carried out, located within the districts of Uttar Pradesh, India. Our hospital, positioned on the border of Shahjahanpur and Lakhimpur Kheri, is marked in white. The customized district (light grey) was developed by merging the hospital’s catchment area with the VCs indicated with dots. The maps were sourced from the Natural Earth (public domain) website (https://www.naturalearthdata.com/downloads/50m-cultural-vectors/50m-admin-1-states-provinces/) and then modified. [Original].

The research was a cross-sectional survey exploring blindness and vision impairment (VI) within the general population. It took place from 29^th^ April to 19^th^ June 2022 across two neighbouring districts in Uttar Pradesh: Lakhimpur Kheri, with roughly four million people, and Shahjahanpur, home to about three million residents. [7] Both districts are mainly rural communities (Fig 1). [7]

We developed a sampling frame that reflected the service area of our organization, which includes our surgical centre located on the border of two districts and its VCs. This frame comprised five blocks from Lakhimpur Kheri (Gola (Kumbhi), Bankeyganj, Mitauli, Mohammadi, Pasgawan) and three blocks from Shahjahanpur (Bhawal Khera, Khutar, Powayan). This tailored ‘district’ encompassed a catchment area of approximately 2·03 million all-age people. [7]

### Inclusion-exclusion criteria

Individuals aged 50 and above who had been ordinarily resident for at least 6 months prior to the survey and who consented were included in the study. Visitors and those unwilling to consent were excluded. In addition, those found to be mentally unsound and unable to provide consent were excluded. This was assessed by the ophthalmologist, who was the clinical lead for each team. This assessment was based on the coherence of responses during the general introductory conversation and the ability to communicate.

### Sample size

The target sample size was 4,092, based on an expected blindness prevalence of 3·5% among individuals aged 50 and older, estimated according to the prevalence of blindness found in a district of Uttar Pradesh from a national survey, which was the most socio-demographically comparable. [4] We included the diabetic retinopathy module in our RAAB survey, and as per convention for such surveys, we used a cluster size of 35. A design effect of 1·4 was used for a cluster size of 35, with a projected 10% non-response rate, a relative precision of 20%, and a 95% confidence level. The population estimate for those aged 50 and older was 406,914, assuming they constitute 20% of the total population of 2,034,568 from the 2011 census, [7] adjusted for the decadal growth rate.

### Sampling

The sample consisted of 117 clusters with 35 individuals aged 50 and older. A two-stage cluster sampling method was used. The details have been reported elsewhere. [8] The team planned to revisit households at the end of the day to include anyone eligible but unavailable during the initial visit.

### Clinical examination

The team presented comprehensive study information and discussed participants’ rights to refuse or withdraw consent, as well as the potential benefits of participation. Written consent was sought from eligible participants. All consenting individuals underwent vision screening and ophthalmic examinations by study team members according to the RAAB protocol. [6] Peek Acuity was used to assess distance vision, measuring uncorrected visual acuity (VA) in both eyes, corrected VA if the participant owned distance spectacles, and pinhole VA in any eye with VA worse than 6/12. Presenting VA was the better of uncorrected or corrected VA based on spectacle use. Any eye with presenting VA worse than 6/12 was examined for the main cause of poor vision, and the principal cause of vision impairment per person was noted, focusing on the more treatable eye. If presenting VA was worse than 6/12 without obvious anterior segment abnormalities, the eye was dilated for posterior segment examination. Minor ocular conditions were referred to the VC, while other conditions were sent to our regional secondary hospital. The optional Diabetic Retinopathy (DR) module was included but will not be described here as detailed findings have been published elsewhere. [8]

### Outcomes

Blindness was defined as presenting VA worse than 3/60 in the better eye. Severe VI was VA of 3/60 or better but worse than 6/60; moderate VI was (MVI) VA of 6/60 or better but worse than 6/18; and mild VI was VA of 6/18 or better but worse than 6/12. [9] Cataract surgical outcome (CSO) was based on the presenting VA post-surgery. Cataract surgical coverage (CSC) was defined as the proportion of those needing surgery who received it; effective CSC was defined as the proportion of those operated on with a good outcome (6/12 or better) among all those needing cataract surgery. [10] Distance refractive error coverage (REC) was the proportion needing glasses (better eye UCVA worse than 6/12 improving to 6/12 with correction or pinhole) who received them; distance effective REC (eREC) was those who received glasses and had a corrected VA of 6/12 or better, out of all those needing glasses. [10]. Relative quality gap (RQG) for refractive error coverage and cataract surgical coverage was calculated as the ratio of the difference between coverage and effective coverage to the coverage

### Data collection – training

For RAAB, each data collection team consisted of four members: an ophthalmologist (team leader), an optometrist, a cluster informer, and a village guide. A qualified RAAB trainer conducted a four-day training session on RAAB procedures, inter-observer variability tests, and fieldwork. Staff were trained to consistently identify eligible participants, assess visual acuity, and conduct lens examinations. Standardised instructions—including definitions, participant selection methods, examination protocols, and data recording techniques—were provided to each team. If a Kappa score of 0·7 was not achieved in the inter-observer variability test, teams received retraining on specific issues and retook the test until standards were met.

### Health system module

Once the RAAB survey was completed, data collection for the RAAB Health System module began.

The module consists of three sections (S1 File):

- A: Policies related to eye health
- B: Socio-demographic characteristics of the population in the sampling area
- C: Health facility data, including availability and volume of service for cataract, refractive error and diabetic retinopathy in 2022

Here, we report results only from section C. The results from Section A and B are included in S2 File.

Our outreach manager was appointed as the module coordinator. The chief medical officers (CMOs) of both districts were contacted to gather information from their offices. For section A related to policy, most data was collected in coordination with the government office. Simultaneously, the rest was obtained from the government’s National Program for Control of Blindness and Visual Impairment (NPCB VI) website by the PI. Data for section B, which covers the sociodemographic characteristics of the population in the sampling area, was extracted from the census by the PI. Data for section C, which covers health facilities, was primarily collected by visiting eye care providers, with the senior administrator at the health facilities being contacted. Data on cataract surgeries performed was also confirmed with the CMO office. Data from health providers operating in the area that have been operational for more than a year and conduct at least 100 cataract surgeries annually were included. The data from our primary eye care centres were included within the hospital data. Data on any services missing from any facility were recorded. The standalone optical shops, being small and scattered across the catchment area with no available data on their existence, were difficult to visit for data collection and were excluded.

### Data management

Survey data was collected using the RAAB7 app on Android devices and synchronised to the RAAB Amazon Web Services (AWS) server in Mumbai when the devices had internet access. The survey coordinator and principal investigator (PI) reviewed the data uploads daily, resolving any discrepancies with the examination teams. RAAB Health Systems module information was recorded in a custom Excel spreadsheet developed for the Health System module.

### Analysis

Automated RAAB7 analysis generated crude and age-sex adjusted prevalence estimates of blindness and VI. The analysis included estimation of CSO, CSC, eCSC, REC, and eREC. [11] Since most participants were from rural areas, with only a small proportion from semi-urban areas, a comparison between urban and rural samples was not performed. P-values were calculated using Pearson’s chi-square test applied to the entire sample. These tests did not account for clustering in variance estimation and should therefore be interpreted as p-values unadjusted for clustering.

The Health System module data were summarised for publication purposes with facility names concealed. Extrapolation to the population aged 50 years and above was carried out using the population projection report released by the government. [12]

### Ethical considerations

The Institutional Review Board of Dr Shroff’s Charity Eye Hospital approved the RAAB study (IRB/2022/JAN/88). Approval for the Health System module implementation was included in the ethics approval for the RAAB7 project from the London School of Hygiene & Tropical Medicine (15504−07). The study adheres to the principles outlined in the Declaration of Helsinki. The names of the health facilities from which data for the health system planning module were collected were not shared for analysis or publication. No patient data was collected from any health facility.

### Informed consent and consent for publication

Written informed consent was obtained in the local language (Hindi) from all participants included in the RAAB survey. For those who were illiterate, a thumbprint was recorded and witnessed by an independent person not affiliated with the study team.

## Results

A total of 3,867 participants out of 4,095 enumerated were examined, resulting in a response rate of 94·4%. The remaining individuals declined to participate. Of those examined, 1,928 (49·8%) were women. The largest number of participants was in the 50–59 years age group (48·9%), followed by 60–69 years (36·0%) and 70–79 years (11·9%).

The age-sex adjusted prevalence of blindness was 2·2% (95% CI 1.7–2·7%) and was higher in women (2·8% [95% CI 2·1–3·4%]) than in men (1·6% [95% CI 1·0–2·1%]) (p < 0·001). The overall prevalence of VI (including mild, moderate, and severe cases) was 29·5%. Details of the prevalence and extrapolated numbers in each category of VI are presented in Table 1. The prevalence of MSVI (moderate or severe VI) was significantly higher in women than in men (21·0% vs 19·9%, p < 0·001).

**Table 1:** Age-adjusted and age-sex adjusted prevalence and extrapolated magnitude of blindness and vision impairment.

	Female			Male			Total		
	%	95% CI	Magnitude	%	95% CI	Magnitude	%	95% CI	Magnitude
**VI threshold**									
Blind	2·8	2·1 - 3·4	4372	1·6	1·0 - 2·1	2516	2·2	1·7 - 2·7	6889
Severe	3·6	2·9 - 4·4	5768	3·5	2·6 - 4·4	5624	3·6	3·0 - 4·2	11392
Moderate	17·3	15·6 - 19·0	27476	16·4	14·6 - 18·1	26238	16·9	15·5 - 18·2	53714
Mild	10·2	8·8 - 11·7	16261	7·6	6·3 - 8·9	12202	9·0	8·0 - 10·0	28464
Total (Any vision loss category)			53877			46580			100459
Total (Aged 50 and above) in the population			158677			160225			318902

### Causes of blindness and vision impairment

Unoperated cataract was identified as the most common cause of blindness (76·5%), severe VI (84·3%), and moderate VI (54·7%) (Table 2). Overall, out of the estimated 100,459 people with blindness or any degree of VI (including mild VI) (Table 1), 41,165 were attributed to unoperated cataract in both eyes (S3 Table). There were projected 23,829 individuals with MSVI or blindness due to cataract in both eyes. An additional 41,984 individuals with cataract in one eye with VA less than 6/18 and 47,840 individuals with VA less than 6/12 were estimated. (S3 Table) in the study region. Uncorrected refractive error was noted as the leading cause of mild VI in the sample (79·2%). Non-trachomatous corneal opacity was the second most common cause of blindness (11·9%). Details of the causes are provided in Table 2. A total of 94·1% (64/68) of blindness and 95·8% (708/739) of MSVI in the survey population were found to be due to avoidable causes (i.e., preventable or treatable).

**Table 2:** Principal causes of blindness and vision impairment among sample participants.

Principal Cause	Blind n, (%)	Severe VI n, (%)	Moderate VI n, (%)	Mild VI n, (%)
Cataract	52, (76·5)	107, (84·3)	335, (54·7)	59, (17·3)
Other corneal opacity	8, (11·8)	4, (3·1)	4, (0·7)	0, (0)
Other posterior segment disease	3, (4·4)	2, (1·6)	14, (2·3)	0, (0)
Cataract surgical complications	1, (1·5)	4, (3·1)	25, (4·1)	8, (2·3)
Glaucoma	1, (1·5)	1, (0·8)	4, (0·7)	1, (0·3)
Refractive error	0, (0)	6, (4·7)	214, (35·0)	270, (79·2)
Others	3, (4·4)	3, (2·3)	16, (2·6)	3,(0·8)
Total	68	127	612	341

### Cataract surgical coverage and distance refractive error coverage

Cataract surgical coverage was 59·6% at a threshold of 6/12 and 70·3% at 6/18. The relative quality gap was 36·6% in both categories (Table 3). The difference in coverage between men and women was not statistically significant. The main barriers to cataract surgery identified in the survey population were: ‘need not felt’ (47·9%), ‘cannot access surgery’ (22·5%), and fear of surgery (9·9%).

**Table 3:** Age-adjusted and age-sex adjusted cataract surgical coverage and distance refractive error coverage.

	Female		Male		Total	
Service coverage indicator	%	95% CI	%	95% CI	%	95% CI
eCSC < 6/12	38·0	34·6 - 41·4	37·6	33·4 - 41·8	37·8	35·0 - 40·6
CSC < 6/12	62·1	58·4 - 65·9	56·3	52·2 - 60·5	59·6	56·5 - 62·7
RQG < 6/12	38·8		33·2		36·6	
eCSC < 6/18	44·1	40·2 - 48·1	45·3	40·2 - 50·4	44·6	41·2 - 48·0
CSC < 6/18	72·1	68·2 - 76·1	67·8	63·2 - 72·4	70·3	67·1 - 73·5
RQG < 6/18	38·8		33·2		36·6	
Distance eREC	7·1	4·0 - 10·3	16·2	11·7 - 20·6	11·2	8·5 - 13·9
Distance REC	9·5	5·8 - 13·3	19·5	14·6 - 24·4	14·0	10·9 - 17·1
RQG	25·3		16·9		20·0	

eCSC = effective cataract surgical coverage; CSC = cataract surgical coverage; RQG = relative quality gap; eREC = effective refractive error coverage; REC = refractive error coverage. The Snellen VA listed in the table (<6/12 and <6/18 pinhole VA) indicates that individuals with worse than that level of pinhole VA were included in the coverage estimates.

Distance refractive error coverage was 14·0%, significantly lower among women (9·5%, 95% CI 5·8–13·3) compared to men (19·5%, 95% CI 14·6–24·4; p < 0·001). The relative quality gap between distance REC and eREC was 20% (Table 3).

### Cataract surgery visual acuity outcomes

Of the 1,119 operated eyes in the sample, 805 (72%) were treated at non-governmental non-profit facilities, while 8% were treated in government facilities. The rest were operated in private for-profit facilities. Overall, 59·8% of the eyes had post-operative presenting visual acuity of 6/12 or better. The proportion of individuals with good outcomes was significantly higher among those operated on within 2 years of the survey compared to those operated on earlier (74·4% vs 57·0%; p < 0·0001). Unaddressed residual refractive error and co-morbidities were identified as the main reasons for not achieving post-operative presenting visual acuity of 6/12 or better. Details of cataract surgical outcomes are provided in Table 4.

**Table 4:** Details of cataract surgical outcomes among sample participants.

Cataract Surgery	Female n, (%)	Male n, (%)	Total n, (%)
Years since surgery (median [IQR])	5 (2 –8)	5 (2 –9)	5 (2 –8)
Operated in government hospitals	59, (9·0)	27, (5·8)	86, (7·6)
Operated in NGO hospitals	468, (71·7)	337, (72·1)	805, (71·9)
**Cataract Surgical Outcomes**			
Good Post-operative outcomes-Presenting visual acuity ≥6/12 (all eyes)	377, (57·8)	292, (62·5)	669, (59·8)
Good Post-operative outcomes -Presenting visual acuity ≥6/12 in eyes operated <2 yrs	75, (74·3)	59, (74·7)	134, (74·4)
Borderline Post-operative outcomes- Presenting visual acuity <6/12–6/60 (all eyes)	217, (33.3)	127, (27.2)	344, (30.7)
Poor Post-operative outcomes-visual acuity <6/60 (all eyes)	58, (8.9)	48, (10.3)	106, (9.5)
Eyes with non-good outcomes- Borderline and Poor outcomes combined (all eyes)	275, (42·2)	175, (37·5)	450, (40·2)
Total eyes operated in the sample (N)	652	467	1119
**Main cause of non-good outcomes (all eyes) %**			
Refractive error	129, (46·9)	69, (39·4)	198, (44·0)
Comorbidities	66, (24·0)	55, (31·4)	121, (26·9)
Posterior Capsular Opacification	46, (16·7)	22, (12·6)	68, (15·1)
Surgical complications	25, (9·1)	20, (11·4)	45, (10·0)
Other long-term sequelae	9, (3·3)	9, (5·1)	18, (4·0)
Total number with non-good outcomes	275	175	450

The results regarding the prevalence of diabetes, prevalence of DR, and the proportion of people with diabetes who have undergone screening for DR have been published earlier.[8]

### Health system module

A total of 14 service providers were found to be eligible, and all were included. These included 10 non-governmental organizations (NGOs), 2 government hospitals, and 2 private for-profit organizations. Permanent cataract services were provided by 10 providers, refraction services by 11, glasses dispensing by 9, and screening and treatment of DR by 2. Data on the number of cataract surgeries were available from 12 providers (86%), but only 1 facility (7%) had pre- and post-operative vision data. Data on the cost of cataract surgery for the patient were available from 9 providers (64%). Data on the number of refractions were available from 7 (50%) providers, and on dispensed glasses from 4 providers (29%) that offer permanent or occasional refractive services. Data on DR screening or treatment were unavailable from all providers. Details on data availability are included in S1 Table.

According to available data (12/14 providers), a total of 14,545 cataract surgeries were performed from January to December 2022, with 3,395 (23%) being phacoemulsification and the rest manual small incision cataract surgery (MSICS). Around 40% of all surgeries (5946 procedures) were done by our hospital. The two government hospitals contributed approximately 4% of the total surgical volume (625 surgeries), while the rest was contributed by non-governmental non-profit organizations. Data from the two private providers, which have relatively low-volume cataract programs (an estimated combined total of fewer than 500 surgeries per year), were not available. Based on the data available, the cataract surgical rate (CSR; the number of cataract surgeries per million all-age people per year) [13] for this region was 7,165 per million population. For the single facility with available post-operative visual acuity data, 87% achieved the best-corrected visual acuity of 6/12 or better at the final follow-up between 4–6 weeks post-surgery, and 2·6% had worse than 6/60. Across 9 of 14 providers (including all three sectors), cataract surgery packages for patients, including consumables (such as intraocular lenses, medications, investigations, and follow-up till 3 months), ranged from free for MSICS to a median of INR 7,000 (US$81·3) for phacoemulsification.

According to data from 7/14 providers, a total of 77,086 refractions were performed, and from 4/14 providers, 21,231 glasses were dispensed in the region. Spectacles were offered at median prices of INR 250 (US$2·9) for ready-made near glasses, INR 500 (US$5.8) for customized single vision glasses, and INR 700 (US$8·1) for bifocals. The number of patients receiving DR screening or treatment could not be estimated due to lack of data. The data collected using the module are available as S2 Table.

## Discussion

We conducted a RAAB survey to evaluate the prevalence and causes of blindness and VI, as well as service indicators in the population, to support planning. [5] Since our secondary hospital was located at the border of two large districts, a custom sampling frame was created from the catchment area to assist service planning. A Health System module was implemented for the first time in the same location and identified gaps in data availability for planning. Tools like the WHO Eye Care Situation Analysis Tool (ECSAT) [14] are already available for situational analysis, but can be resource-intensive to use and are intended for national-level reporting. Because RAABs are mainly conducted at the subnational level to plan services, a quick-to-implement tool that supports eye care planning within a survey area can add significant value.

We estimated that approximately 100,000 people aged 50 and above have mild VI or worse, with more than 40,000 cases caused by untreated cataract in the catchment. We observed a slightly higher prevalence of blindness and VI in the study area compared to national estimates. [4] In the national RAAB, the prevalence was higher among people living in rural areas, and our predominantly rural catchment area may have contributed to this increased prevalence. Similar to the national RAAB findings, unoperated cataract and corneal opacities were the two most common causes of blindness. However, unlike the national RAAB, where cataract surgical complications accounted for 7·2% of blindness, in our survey region, they made up only about 2%. This aligns with the finding that 60% of participants operated on for cataract had good post-operative VA, and surgical complications were responsible for only 10% of poor outcomes. Among those who had surgery less than two years before the survey, 74% had a good post-operative VA; this is an encouraging trend and increases confidence that scaling up surgical volume could be prioritized. This trend towards better outcomes could be due to improved infrastructure, better-trained human resources, including surgeons, and a higher proportion of patients undergoing phacoemulsification, resulting in better uncorrected visual acuity. However, collecting cataract surgical facility data on trends in these factors was beyond the scope of this study.

Corneal opacities were the second leading cause of blindness. Komal et al. have reported the effectiveness of VCs equipped with tele-ophthalmology in managing patients with corneal conditions. [15] All our VCs now offer tele-ophthalmology services with a cornea specialist available at the tele-ophthalmology hub located at the tertiary hospital in Delhi. This enables definitive decision-making regarding the need for referral to the nearest surgical centre at the secondary level. An ophthalmologist trained in cornea care is stationed at the secondary-level referral hospital, enabling local treatment. Although this improves the infrastructure for early intervention for corneal conditions among patients presenting to the health system, more needs to be done to promote prevention and encourage early presentations. In this predominantly rural region [7], agricultural trauma could be an important cause of corneal infections and blindness. [16] Targeted activities to prevent agricultural injuries, raise awareness of injury prevention, and emphasise the need to seek care early after an injury should be planned.

In the study region, there were two government hospitals, two for-profit private hospitals, and the remaining ten service providers were non-profit, non-governmental organizations. Excluding data related to pre- and post-operative VA and DR, which were missing for nearly all facilities, the availability of data was notably better from government and NGO compared to private providers. This could also be due to the reluctance of the for-profit providers to share their data, as some of it could be used to estimate their income.

CSC and eCSC at a cut-off of 6/12 were 59·6% and 38%. These closely matched the national estimates of 57·3% (95% CI 53·3–61·2%) and 36·7% (95% CI: 33·6, 39·9), respectively. eCSC was very similar to that reported in the national survey [17] and in the study by Ramke et al., which used datasets from 20 countries, [18] but lower than the median for the South-East Asia region in a more recent study by McCormick et al., based on population-based surveys from 55 countries. [19] The relative quality gap [10] of 36·6% was also comparable to the national average of 36%.[16] Women had higher CSC but also higher quality gap at VA < 6/18 and 6/12. This difference could have been due to poor spectacle coverage among women as even the proportion of women with non-good cataract outcome due to uncorrected refractive error (46·9%) was higher among women than among men (39·4%). The backlog of cataract surgeries for eyes with VA less than 6/12 (the cut-off used by WHO for eCSC) was approximately 130,000, with about 41,000 individuals needing surgery in both eyes (82,000 eyes) and almost 48,000 in one eye (S3 Table). This was available from the RAAB7 report. Since data on the number of cataract surgeries was unavailable from only two providers, both with relatively low-volume cataract programs, we believe that the CSR of the region would be very close to our estimate of 7,165 per million population per year (14,545 surgeries in a population of 2·03 million). Although this figure was much higher than the national average of 5,900 in 2022, [20] there remains a significant need to increase surgical volume in the catchment area.

This situational analysis helped us plan services for our health facility. At the time, only about 14,500 cataract surgeries were performed annually despite a significant unmet need. Only 8% of the individuals found in the survey who had undergone cataract surgery had the surgery at governmental facilities. This aligned well with the data collected in the Health System module, which showed that the contribution of the two government providers was 4% in the most recent calendar year. Conversely, 72% of the operated patients had their surgery at NGO facilities. According to data collected for the Health System module, these organizations contributed around 95% of the surgical volume in the region. This highlights the important role of these non-governmental organizations in reducing cataract-related vision loss in the study region. Our organization expanded its infrastructure and staff, increasing cataract surgeries from fewer than 6000 in 2022 to over 16,000 in 2024, while maintaining good outcomes. Forty percent of the region’s cataract surgeries with available data (5946 out of 14,545) were performed by our organization, so scaling up our services is likely to increase overall coverage in the region.

eREC among people aged 50 years and older was very low at 11%, and even lower among women (7%). eREC was much lower than that reported in a study from southern India by Subburaman et al., where eREC declined with age [21]. That study area had 7 VCs and 1 clinic for about 1 million people, while a similar number of centres served twice as many people in our area. Since refraction and dispensing are key functions of VCs [22^],^ better access might explain the difference. Health-seeking behaviour, not assessed in our study, could also have contributed to this difference.

About 21,000 glasses were dispensed by 4 of the 14 secondary level hospitals in the area; however, most secondary level providers did not have data available and our primary level vision centres and individual optical shops could not be surveyed for logistical reasons, resulting in incomplete data. This may have resulted in an underestimation of the glasses dispensed, as Marmamula et al.‘s study among the rural population in southern India reported that up to 70% of glasses were obtained from optical shops. [23] However, even considering these figures, the totals are expected to be significantly lower than the estimated need of around 14%, as reported in a case study from Aravind. [24] Between 2022 and 2024, we increased the number of VCs from 7 to 24 in this region to improve access, especially for women, who are more likely to use likely to use primary care centres [25] but the need for refractive error correction in the population younger than 50 years, and the extent to which it is being met, are unknown and require further study.

One significant gap across facilities was the lack of pre-operative visual acuity data for both eyes, which is essential for categorizing patients into different levels of vision impairment. There was also limited access to post-operative visual acuity data. Although this data was available on the government portal, it was only for patients whose providers claimed reimbursements through government schemes under national programs. Data on the screening or treatment of DR were not maintained by any of the organizations in the area. This was a major gap in data availability identified by our study using the health system module. One recommendation from this study is to establish a national portal where organizations could be encouraged to share their cataract surgical outcomes without revealing their own or the patients’ identities. Data from such a portal could help estimate trends in the number and quality of surgeries in a region without the need to repeat RAAB surveys. The Government of India’s initiative to create a unique ID for each patient on a shared portal could be an important first step toward enhancing data availability. [26] This platform would be accessible to all registered providers with a unique ID for each patient. Such a platform would also be useful for estimating the coverage of DR screening in the region and for recalling people with diabetes for regular retinal check-ups. Before that, better systems for capturing data at the facility level could be developed. We are also updating our electronic records to allow entry of relevant data for people with diabetes and refractive errors via a drop-down menu, improving data extraction capabilities for these services. A few years back, a similar national-level initiative to capture cataract outcomes was attempted [27] but was unsuccessful. This highlights that the issue is not just technical but also behavioural and systemic. Research into strategies to encourage behavioural change and adoption is required.

One limitation of this study was that, as a non-governmental organization, we could not access the available post-operative visual acuity data due to confidentiality restrictions. We also could not include information on refraction and glasses sold from optical shops due to logistical reasons. Another limitation of this study was that RAAB includes only individuals aged 50 years and above, so even with good refraction data, it would not be possible to relate refraction data from facilities to the eREC in the population aged 50 and above, as obtained from RAAB.

One limitation of the health system module is that it is intended to collect information only on cataract, refractive error, and DR. However, these are the most common causes of vision loss, especially in low- and middle-income countries, so we feel it captures most of the information required for regional planning. Restricting the module to common conditions also makes it relatively rapid and less resource-intensive.

Our study had several strengths. It presents the first real-world application of this new Health System Module tested in a large, mainly rural service catchment in North India. For cataract surgery volume, the analysis shows that integrating robust epidemiological data with service capability profiling can uncover gaps that remain hidden when either data type is analysed separately. However, due to a lack of data on cataract outcomes, refractive errors and DR, the same conclusion cannot be drawn for the assessment of the quality of cataract surgery delivery and the delivery of services for other eye health conditions.

One notable strength was the excellent response rate of over 94%. Additionally, it was among the first RAAB surveys to use RAAB7 [6] for mobile data collection with built-in logic that reduces data entry errors and enables real-time monitoring. It was also the first study to conduct a RAAB survey in a region tailored to a service provider, and to perform a situational analysis using the Health System module.

A study by Randrianaivo et al. combined a population-based survey with a limited assessment of cataract services in the region, [28]; we could not find any study where a population-based assessment of the burden of visual impairment was integrated with a structured and comprehensive evaluation of eye care services in the same region.

While RAAB with the DR module identified population needs, the Health System module helped assess data and service gaps. Using the tool, we estimated various elements of service provision for the region, including the number of annual cataract surgeries, refractions, and the prices of surgeries and glasses, and related those to the regional needs.

Although the Health System Module provides the capacity to collect comprehensive data on the three eye conditions, our study highlights both its feasibility and operational limitations. Data completeness at the facility level, the feasibility of collecting data from small optical shops, reservations about sharing outcome data from private providers, and the lack of open-source outcome data are some of the challenges highlighted by our study. All these can reduce the programmatic utility of the Health System Module.

## Conclusions

Our study introduced a new model for utilizing RAAB and the Health System module to enable a more comprehensive situational analysis from a regional provider’s perspective. Since RAAB is the most widely used population-based eye health survey method worldwide, this extension can be broadly adopted by planners, governments, and organizations to strengthen eye care systems and reduce blindness and vision impairment in their catchment areas. Additionally, the module can identify gaps in the data required for regional planning. Its usefulness can be increased through improved data collection at the facility level and by improving the feasibility of data sharing.

## Supporting information

S1 Table Service provider data availability for the Health System module. (DOCX)

S2 Table Results of Section C of Health System Planning Module. (DOCX)

S3 Table Details of Individuals with Bilateral And Unilateral Visual Acuity Less Than 6/12 Due To Cataract. (DOCX)

S1 File Complete Template of the Health System Module. (XLSX)

S2 File Filled Sections A and B of the Health System Module. (XLSX)
