# Supplementary material for: Beyond prevalence: A complete catchment eye health system profile using rapid assessment of avoidable blindness, a population-based eye health survey, and the first implementation of the newly developed health system module
Source: PLoS One. 2026 Aug 10;21(8):e0354744. doi: 10.1371/journal.pone.0354744 (PMC13456199; doi:10.1371/journal.pone.0354744)
Supplement: Supplementary file 1 [file pone.0354744.s001.docx]

Supplementary Tables S1

S1 Table. **Service provider data availability for the Health System module**

|  | **Type of facility** | | | | | |  | |
| --- | --- | --- | --- | --- | --- | --- | --- | --- |
|  | **Government (n=-2)** | | **NGO (n=10)** | | **Private for profit (n=2)** | | **All (n=14)** | |
| **Service Characteristics** | **N** | **%** | **N** | **%** | **N** | **%** | **N** | **%** |
| **Cataract Services** |  |  |  |  |  |  |  |  |
| Service Availability (Permanent or occasional) | 2 | 100 | 10 | 100 | 2 | 100 | 14 | 100 |
| Data Availability of cataract services |  |  |  |  |  |  |  |  |
| Waiting time (days) | 2 | 100 | 10 | 100 | 0 | 0 | 12 | 86 |
| Pre-op VA | 0 | 0 | 1 | 10 | 0 | 0 | 1 | 7 |
| Number of surgeries |  |  |  |  |  |  |  |  |
| Phaco/MSICS | 2 | 100 | 10 | 100 | 0 | 0 | 12 | 86 |
| Post-op VA | 0 | 0 | 1 | 10 | 0 | 0 | 1 | 7 |
| Cost of surgery | 2 | 100 | 5 | 50 | 2 | 100 | 9 | 64 |
| **Refractive services** |  |  |  |  |  |  |  |  |
| Service Availability (Permanent or occasional) | 2 | 100 | 10 | 100 | 2 | 100 | 14 | 100 |
| Data Availability of refractive services |  |  |  |  |  |  |  |  |
| Number of refractions | 2 | 100 | 5 | 50 | 0 | 0 | 7 | 50 |
| Spectacles dispensed | 2 | 100 | 2 | 25 | 0 | 0 | 4 | 29 |
| **Diabetic Retinopathy Services** |  |  |  |  |  |  |  |  |
| Service Availability for Screening (Permanent or occasional) | 2 | 100 | 6 | 60 | 2 | 100 | 10 | 71 |
| Service Availability for Treatment (Permanent or occasional) | 0 | 0 | 2 | 20 | 2 | 100 | 4 | 29 |
| Data Availability of DR services |  |  |  |  |  |  |  |  |
| Number of Screening or treatment | 0 | 0 | 0 | 0 | 0 | 0 | 0 | 0 |
