# Supplementary material for: Beyond prevalence: A complete catchment eye health system profile using rapid assessment of avoidable blindness, a population-based eye health survey, and the first implementation of the newly developed health system module
Source: PLoS One. 2026 Aug 10;21(8):e0354744. doi: 10.1371/journal.pone.0354744 (PMC13456199; doi:10.1371/journal.pone.0354744)
Supplement: Supplementary file 2 [file pone.0354744.s002.docx]

Supplementary Tables S2

**S2 Table. Results of Health System Planning Module**

|  | **Provider type** | | | |  |
| --- | --- | --- | --- | --- | --- |
|  | **Government** | **NGO** | **Private** | **All** |  |
| **CATARACT SERVICES** |  |  |  |  |  |
| **Number of facilities** |  |  |  |  |  |
| Permanent | 2 | 6 | 2 | 10 |  |
| Occasional | 0 | 4 | 0 | 4 |  |
| Not Available | 0 | 0 | 0 | 0 |  |
| **Results** |  |  |  |  |  |
| Waiting time | 0-4 weeks | 0-2 weeks | NA |  |  |
| Pre-op VA (in the operated eye) |  |  |  |  |  |
| Normal |  | 139, (12.6%) |  |  | Only our facility data for 5353 patients |
| Mild |  | 123, (2.3%) |  |  |  |
| Moderate |  | 1187, (22.2%) |  |  |  |
| Severe |  | 932, (17.4%) |  |  |  |
| Blind |  | 2972, (55.5%) |  |  |  |
| Number of surgeries |  |  |  |  |  |
| Phaco | 0 | 3395, (24.4%) | NA | 3,395 |  |
| SICS | 625, (100%) | 10521, (75.6%) | NA | 11,146 |  |
| Other |  |  |  |  |  |
| Post-op VA |  |  |  |  |  |
| Normal |  | 3763, (86.6%) |  |  | Only our facility data, Data of the surgeries for which government reimbursement is asked is with the district office but not shared due to data privacy |
| Mild |  | 250, (5.8%) |  |  |  |
| Moderate |  | 218, (5.0%) |  |  |  |
| Severe |  | 43, (1.0%) |  |  |  |
| Blind |  | 71, (1.6%) |  |  |  |
| Cost |  |  |  |  |  |
| Phaco | NA | 4000-22,000 | 8000- 35,000 |  |  |
| SICS | 0 | 0-5000 | 4000-8000 |  |  |
| **REFRACTIVE ERROR** |  |  |  |  |  |
| **Number of facilities providing ~~R~~efraction** |  |  |  |  |  |
| Permanent | 2 | 7 | 2 | 11 |  |
| Occasional | 0 | 3 | 0 | 3 |  |
| Not Available |  |  |  |  |  |
| **Number of facilities providing spectacles dispensing ~~services~~** |  |  |  |  |  |
| Permanent | 0 | 7 | 2 |  |  |
| Occasional | 2 | 0 | 0 |  |  |
| Not Available | 0 | 3 | 0 |  |  |
| Refraction | 50304 | 26782 | NA | 77086 |  |
|  |  |  |  |  |  |
| Spectacles dispensed | 11883 | 9348 | NA | 21,231 | Data available from only 2 NGO facilities and the 2 government facilities |
|  |  |  |  |  |  |
| **DR SERVICES** |  |  |  |  |  |
| **Number of facilities providing Screening** |  |  |  |  |  |
| Permanent | 0 | 2 | 0 | 2 |  |
| Occasional | 2 | 4 | 2 | 8 |  |
| Not Available | 0 | 4 | 0 | 4 |  |
| **Number of facilities providing Treatment** |  |  |  |  |  |
| Permanent |  | 2 |  | 2 |  |
| Occasional |  | 0 |  | 0 |  |
| Not Available | 0 | 0 | 0 | 0 |  |
| **Patients screened/ treated** | no data available | | | | |
