# Supplementary material for: Beyond prevalence: A complete catchment eye health system profile using rapid assessment of avoidable blindness, a population-based eye health survey, and the first implementation of the newly developed health system module
Source: PLoS One. 2026 Aug 10;21(8):e0354744. doi: 10.1371/journal.pone.0354744 (PMC13456199; doi:10.1371/journal.pone.0354744)
Supplement: Supplementary file 3 [file pone.0354744.s003.docx]

Supplementary Table S3

**Supplementary Table 3. Details of Individuals with Bilateral And Unilateral Visual Acuity Less Than 6/12 Due To Cataract**

|  | Female | | | Male | | | Total | | |
| --- | --- | --- | --- | --- | --- | --- | --- | --- | --- |
| VI threshold | % | 95% CI | Extrapolated magnitude | % | 95% CI | Extrapolated magnitude | % | 95% CI | Extrapolated magnitude |
| Bilateral |  |  |  |  |  |  |  |  |  |
| PinVA <3/60 | 1.1 | 0.7 - 1.5 | 1763 | 0.9 | 0.5 - 1.2 | 1406 | 0.9 | 0.7 - 1.2 | 2914 |
| PinVA <6/60 | 2.7 | 2.1 - 3.3 | 4295 | 1.8 | 1.2 - 2.3 | 2857 | 2.2 | 1.7 - 2.6 | 6896 |
| PinVA <6/18 | 7.7 | 6.5 - 8.8 | 12184 | 7.7 | 6.5 - 8.9 | 12292 | 7.5 | 6.6 - 8.4 | 23829 |
| PinVA <6/12 | 13.1 | 11.6 - 14.6 | 20857 | 13.4 | 11.9 - 15.0 | 21519 | 12.9 | 11.7 - 14.2 | 41165 |
| Unilateral |  |  |  |  |  |  |  |  |  |
| PinVA <3/60 | 6.7 | 5.7 - 7.8 | 10710 | 4.3 | 3.3 - 5.3 | 6941 | 5.6 | 4.8 - 6.4 | 17651 |
| PinVA <6/60 | 8.5 | 7.2 - 9.9 | 13547 | 6.2 | 5.1 - 7.3 | 9913 | 7.4 | 6.5 - 8.3 | 23460 |
| PinVA <6/18 | 14.6 | 13.0 - 16.2 | 23105 | 11.8 | 10.2 - 13.3 | 18879 | 13.2 | 12.1 - 14.4 | 41984 |
| PinVA <6/12 | 16.2 | 14.7 - 17.8 | 25766 | 13.8 | 12.2 - 15.4 | 22074 | 15.1 | 13.9 - 16.3 | 47840 |
