# Supplementary material for: Beyond prevalence: A complete catchment eye health system profile using rapid assessment of avoidable blindness, a population-based eye health survey, and the first implementation of the newly developed health system module
Source: PLoS One. 2026 Aug 10;21(8):e0354744. doi: 10.1371/journal.pone.0354744 (PMC13456199; doi:10.1371/journal.pone.0354744)
Supplement: Supplementary file 6 [file pone.0354744.s008.docx]

Additional Editor Comments :

Based on the revised version, in abstract, modify the conclusion as "Integrating RAAB with a structured health system assessment enabled a unique, comprehensive situational analysis linking epidemiological needs with service capacity. It also highlighted data gaps, feasibility and operational limitations regarding data for cataract, refractive, and diabetic retinopathy services in the region. This model can guide regional planning and strengthen monitoring of effective coverage. However, challenges in data collection can reduce programmatic utility of the Health System Module"

Response:

Thanks for your feedback. We have modified the conclusion in the abstract now to-

### “Conclusion

Integrating RAAB with a structured health system assessment enabled a unique, comprehensive situational analysis linking epidemiological needs with service capacity. It also highlighted data gaps, feasibility and operational limitations regarding data for cataract, refractive, and diabetic retinopathy services in the region. This model can guide regional planning and strengthen monitoring of effective coverage. However, challenges in data collection can reduce the programmatic utility of the Health System Module.”
