# Supplementary material for: Beyond prevalence: A complete catchment eye health system profile using rapid assessment of avoidable blindness, a population-based eye health survey, and the first implementation of the newly developed health system module
Source: PLoS One. 2026 Aug 10;21(8):e0354744. doi: 10.1371/journal.pone.0354744 (PMC13456199; doi:10.1371/journal.pone.0354744)
Supplement: Supplementary file 7 [file pone.0354744.s006.docx]

Response to comments

Our responses are shown in blue font, and the page numbers refer to the revised clean copy.

While Reviewer 1 has recommended rejection, based on the feedback from the other reviewer, we will provide the authors an opportunity to address all the comments. Overall, my observation is that the only new information presented in this paper pertains to the Health System Module, specifically covering areas related to policies, sociodemographic characteristics of the population, and health facility data. This appears to be largely similar to the WHO ECSAT tool. However, the authors have not presented the complete data related to the Health System Module.

Response: Thanks for your comments and this opportunity. In the manuscript, we have included the results from the section with health facility data. However, we have now added the results of the other two sections as supplementary files. Although this tool is similar to the ECSAT tool, this can be deployed more rapidly and is less resource-intensive. WHO ECSAT is generally deployed at the national level, whereas this tool is intended for the sub-national level. As RAABs are generally implemented at the sub-national level, this tool could supplement RAAB data for planning services in the region of the RAAB survey. We have highlighted this difference in the ‘Discussion section (lines 298-302)

Additionally, it is well established that the prevalence of blindness and visual impairment is directly correlated with the cataract surgical rate (CSR) of a country or district. In this context, it is unclear what additional, novel information is generated from the Health System Module that has practical or programmatic implications.

Thanks for your comment. Information generated by the Health System Module includes the availability of existing eye care services in the sub-national region under study. This tool is not intended to comment on the prevalence of blindness or visual impairment. It provides information on the prevailing eye care services at the facility level in the region, and this information can supplement the prevalence data collected from surveys like RAAB to plan services based on needs and gaps. Thus, we believe this has programmatic implications.

In our study, it was shown that, despite the lead author’s facility being the primary service provider, other providers were involved. Thus, data from all such facilities, along with data on need in this region from the RAAB study, were used for planning services. (Lines 343-348). In lines 408-411, we mentioned that this model helped estimate the service gap using prevalence and facility data.

Furthermore, the authors need to clarify how information from the Health System Module can be used longitudinally to monitor trends in blindness and visual impairment in a given region.

Information from Health System Module is not related to the prevalence of blindness and visual impairment, and thus trends in blindness and visual impairment cannot be monitored using this. However, as Health System Module can be repeated every few years, trends in overall service provision in the region can be estimated.

Please describe in your methods section how capacity to provide consent was determined for the participants in this study. Please also state whether your ethics committee or IRB approved this consent procedure. If you did not assess capacity to consent please briefly outline why this was not necessary in this case.

Thanks for your comment. We have added the exclusion criteria that were approved by our ethics committee and followed (Line 100-104)

“Individuals aged 50 and above who had been ordinarily resident for at least 6 months prior to the survey and who consented were included in the study. Visitors and those unwilling to consent were excluded. In addition, those found to be mentally unsound and unable to provide consent were excluded. This was assessed by the ophthalmologist, who was the clinical lead for each team. This assessment was based on the coherence of responses during the general introductory conversation and the ability to communicate.”

3. Please include a complete copy of PLOS’ questionnaire on inclusivity in global research in your revised manuscript. Our policy for research in this area aims to improve transparency in the reporting of research performed outside of researchers’ own country or community. The policy applies to researchers who have travelled to a different country to conduct research, research with Indigenous populations or their lands, and research on cultural artefacts. The questionnaire can also be requested at the journal’s discretion for any other submissions, even if these conditions are not met. Please find more information on the policy and a link to download a blank copy of the questionnaire here: <https://journals.plos.org/plosone/s/best-practices-in-research-reporting>. Please upload a completed version of your questionnaire as Supporting Information when you resubmit your manuscript.

Filled and uploaded

4. Thank you for stating the following in the Acknowledgments Section of your manuscript:

[Co-authors on this project are supported by the National Institute for Health Research (NIHR) (using the UK’s Official Development Assistance (ODA) Funding) and Wellcome [215633/Z/19/Z] under the NIHR-Wellcome Partnership for Global Health Research. The views expressed are those of the authors and not necessarily those of Wellcome, the NIHR or the Department of Health and Social Care]

We note that you have provided funding information that is not currently declared in your Funding Statement. However, funding information should not appear in the Acknowledgements section or other areas of your manuscript. We will only publish funding information present in the Funding Statement section of the online submission form.

Please remove any funding-related text from the manuscript and let us know how you would like to update your Funding Statement. Currently, your Funding Statement reads as follows:

[The author(s) received no specific funding for this work.]

Please include your amended statements within your cover letter; we will change the online submission form on your behalf.

No specific funding for this research was received from the sources mentioned in the acknowledgement. We had included those statements because some of the co-authors were supported by the grants mentioned. We would like to seek editorial approval to include those statement and have removed that from the manuscript.

As no specific funding was received by the authors for this study, our Funding Statement remains the same

5. Please note that your Data Availability Statement is currently missing the repository name and/or the DOI/accession number of each dataset. If your manuscript is accepted for publication, you will be asked to provide these details on a very short timeline. We therefore suggest that you provide this information now, though we will not hold up the peer review process if you are unable.

We have included that now (lines 536)

<https://www.doi.org/10.17037/RAABDATASET.b871319b-08ee-4508-bd41-413733c51075>

6. Your ethics statement should only appear in the Methods section of your manuscript. If your ethics statement is written in any section besides the Methods, please delete it from any other section.

We have done this now

7. Please remove your figures from within your manuscript file, leaving only the individual TIFF/EPS image files, uploaded separately. These will be automatically included in the reviewers’ PDF.

We have removed the figure now

8. We note that Figure 1 in your submission contains map images which may be copyrighted. All PLOS content is published under the Creative Commons Attribution License (CC BY 4.0), which means that the manuscript, images, and Supporting Information files will be freely available online, and any third party is permitted to access, download, copy, distribute, and use these materials in any way, even commercially, with proper attribution. For these reasons, we cannot publish previously copyrighted maps or satellite images created using proprietary data, such as Google software (Google Maps, Street View, and Earth). For more information, see our copyright guidelines: <http://journals.plos.org/plosone/s/licenses-and-copyright>.

We require you to either (1) present written permission from the copyright holder to publish these figures specifically under the CC BY 4.0 license, or (2) remove the figures from your submission:

1. You may seek permission from the original copyright holder of Figure 1 to publish the content specifically under the CC BY 4.0 license.

We recommend that you contact the original copyright holder with the Content Permission Form (<http://journals.plos.org/plosone/s/file?id=7c09/content-permission-form.pdf>) and the following text:

“I request permission for the open-access journal PLOS ONE to publish XXX under the Creative Commons Attribution License (CCAL) CC BY 4.0 (<http://creativecommons.org/licenses/by/4.0/>). Please be aware that this license allows unrestricted use and distribution, even commercially, by third parties. Please reply and provide explicit written permission to publish XXX under a CC BY license and complete the attached form.”

Please upload the completed Content Permission Form or other proof of granted permissions as an "Other" file with your submission.

In the figure caption of the copyrighted figure, please include the following text: “Reprinted from [ref] under a CC BY license, with permission from [name of publisher], original copyright [original copyright year].”

2. If you are unable to obtain permission from the original copyright holder to publish these figures under the CC BY 4.0 license or if the copyright holder’s requirements are incompatible with the CC BY 4.0 license, please either i) remove the figure or ii) supply a replacement figure that complies with the CC BY 4.0 license. Please check copyright information on all replacement figures and update the figure caption with source information. If applicable, please specify in the figure caption text when a figure is similar but not identical to the original image and is therefore for illustrative purposes only.

The following resources for replacing copyrighted map figures may be helpful:

USGS National Map Viewer (public domain): <http://viewer.nationalmap.gov/viewer/>
The Gateway to Astronaut Photography of Earth (public domain): <http://eol.jsc.nasa.gov/sseop/clickmap/>
Maps at the CIA (public domain): <https://www.cia.gov/library/publications/the-world-factbook/index.html> and [https://www.cia.gov/library/publications/cia-maps-publications/index.html](https://track.editorialmanager.com/CL0/https:%2F%2Fwww.cia.gov%2Flibrary%2Fpublications%2Fcia-maps-publications%2Findex.html/1/010f019c4061fdbe-911c310f-5bce-48f4-8dcf-39b22a644a76-000000/af6n0Dhy1dISvOBTXgCOEUdSafU7Ld83SmDO3uIKrcw=249)
NASA Earth Observatory (public domain): <http://earthobservatory.nasa.gov/>
Landsat: <http://landsat.visibleearth.nasa.gov/>
USGS EROS (Earth Resources Observatory and Science (EROS) Center) (public domain): [http://eros.usgs.gov/#](http://eros.usgs.gov/)
Natural Earth (public domain): <http://www.naturalearthdata.com/>

Thanks for your feedback. For the revised figure, we have sourced maps from the Natural Earth (public domain) website (<https://www.naturalearthdata.com/downloads/50m-cultural-vectors/50m-admin-1-states-provinces/>) and then modified them.

9. Please include captions for your Supporting Information files at the end of your manuscript, and update any in-text citations to match accordingly. Please see our Supporting Information guidelines for more – see how to add caption

information: [http://journals.plos.org/plosone/s/supporting-information](http://track.editorialmanager.com/CL0/http:%2F%2Fjournals.plos.org%2Fplosone%2Fs%2Fsupporting-information/1/010f019c4061fdbe-911c310f-5bce-48f4-8dcf-39b22a644a76-000000/_nKLa2AvP-md9SocF_G4cBRHp1-YJkwYyEseFn4_Vy8=249).

Thanks, we have done this now (Lines 577-581)

If the reviewer comments include a recommendation to cite specific previously published works, please review and evaluate these publications to determine whether they are relevant and should be cited. There is no requirement to cite these works unless the editor has indicated otherwise.

.

5. Review Comments to the Author

Please use the space provided to explain your answers to the questions above. You may also include additional comments for the author, including concerns about dual publication, research ethics, or publication ethics. (Please upload your review as an attachment if it exceeds 20,000 characters)

Reviewer #1: I appreciate the hard work put into amalgamating the findings of the RAAB survey and the limited analysis of the situation of eye care services in the study area.
The manuscript is well written. However, it has limited value for planning eye health care programs at the primary, secondary, and tertiary levels and covers preventive, curative, and rehabilitative components of eye health. It may send wrong messages to planners of developing countries to blindly use the outcome of the present study to strengthen eye services.

Thanks for your comments. Based on global eye health stakeholder feedback (pending publication), this health system module prioritises three common causes of vision loss: cataract, uncorrected refractive error, and diabetic retinopathy. We also believe that restricting the module to common conditions also makes it relatively rapid and less resource-intensive.

We believe that the information in this module can effectively supplement epidemiological information obtained from RAAB, which is also generally used for planning purposes. However, we have acknowledged as a limitation in the discussion section that only three common eye conditions are included in this module. (lines 403-407)

Reviewer #2: Congratulations to the authors for preparing a manuscript that is highly relevant for eye health planning in the region. The integration of the health system module into RAAB adds significant value by enabling an assessment of current service capacity, which can serve as an important input for effective eye care planning.

Overall, the manuscript is well written. The use of well-established study protocols and the high response rate reflect the systematic execution of the survey. The results are clearly presented based on outputs generated from the RAAB software, and the manuscript covers the major indicators related to cataract services and distance refractive error.

However, I have the following queries and requests for clarification:

1. Line 268: The proportion of individuals with good outcomes was significantly higher among those operated on within two years of the survey compared to those operated on earlier (74.4% vs 57.0%; p < 0.0001).

It would be helpful if the authors could discuss potential factors underlying this difference in visual acuity outcomes, such as changes in refractive error over time or the manifestation of other ocular comorbidities.

Thanks for this comments, for bringing this out. We have added some potential factors now (line 314-318)

“This trend towards better outcomes could be due to improved infrastructure, better-trained human resources, including surgeons, and a higher proportion of patients undergoing phacoemulsification with better uncorrected visual acuity. However, collecting cataract surgical facility data on trends in these factors was beyond the scope of this study.”

2. Table 1: Please clarify how the figure 208,929 was derived. This number does not appear to align with the population details provided. Kindly specify the denominator used to arrive at this estimate.

Thank you for identifying and highlighting this.

Following the completion of the study, we used the age distribution estimates as per the government projection report on population. (16%) <https://nhm.gov.in/New_Updates_2018/Report_Population_Projection_2019.pdf>,

for extrapolation to the population. (We have cited that now-reference 12). Due to a data entry error, the age-gender population group counts were double-counted. We have now corrected that, and the estimated number of individuals aged 50 and above is 318,902 (the new denominator for extrapolating the prevalence of blindness and visual impairment from the results of RAAB in that population; the denominator is now included in the last row of Table 1). Using that as the new denominator, the number of individuals with blindness or any degree of visual impairment (mild, moderate, or severe) was found to be 100459 (adding the estimated number of individuals with blindness, mild, moderate, or severe visual impairment- 6,889 + 11,392 + 53,714 + 28,464). So, the figure 208929 has been replaced with 100459 in Table 1.

3. Table 3: The reported distance effective refractive error coverage (eREC) appears to be considerably lower compared to a recent publication in the AJOI based on a study conducted in Theni district, which reported more than double this rate. The authors may consider including this reference and briefly discussing possible reasons for the observed difference.

Thanks for this comment. We acknowledge that the eREC was much lower than that detected in the study cited by the reviewer. Even in that study, the eREC declined with age. However, as mentioned in that article, there were 7 primary eye centres and 1 city clinic for a population of about 1 million in that region. In comparison, a similar number of primary eye care centres were present in the study area, serving a population twice as large. As one of the major functions of the primary eye care centres is dispensing glasses, better access to these centres could have been one of the reasons for this difference. Health-seeking behaviour, which was not assessed for uncorrected refractive error in our study, could also have contributed to this difference. We have cited this study and added the probable reasons for this difference in the discussion section now (Line 363-367).

4. Line 237: Please clarify how the figure 85,394 was calculated.

The overall population of the study region is reported as 2,034,568, with individuals aged ≥50 years constituting 20% of the population estimated as 406,974. However, most of the rates are relating to a population of 656,000+.

Thanks for highlighting this.

Based on our previous experience working in the catchment district, we used an estimate of 20% of the population aged 50 and above to calculate the sample size. However, to extrapolate the results to the population, we used the detailed age-group and gender-wise projections for the entire state in the government report. That report projected 16% for this age group in Uttar Pradesh <https://nhm.gov.in/New_Updates_2018/Report_Population_Projection_2019.pdf> (We have cited that now-reference 12). Due to a data entry error, the age-gender population group counts were double-counted. Now we have corrected that, and the denominator is 318,902 for people aged 50 and above. This denominator is now mentioned in Table 1. The figure of 85,394 has now been corrected to 41,165 (line 223). This is the number of individuals aged 50 and above, estimated to have any degree of visual impairment due to cataract. This is obtained from the automated report from the RAAB 7 platform, and the table is included as a supplementary table (S2_table.word). We would like to thank the reviewer for this comment, which led to this correction in extrapolating the results to the population.

Additionally, as the rates presented are based on the primary cause of blindness, the actual cataract burden may be higher than the proportions reported in Table 2. The authors may wish to clarify or comment on this aspect.

Thanks for this comment. We agree that the actual cataract load may be higher in the population. Table-2 describes the proportion of people with blindness or visual impairment who have that condition primarily due to cataract. It isn’t trying to estimate the burden of operable cataract, which would be higher than this, as suggested by you. There may have been other individuals with operable cataract but who were not visually impaired. Based on the RAAB report, we have included this in the discussion section by adding the estimated number of eyes with vision less than 6/12 due to cataract (WHO cut-off) among those with unilateral and bilateral cataracts. (lines 341-343)

Given that RAAB has been updated regularly and that some analytical issues have been resolved in newer versions, the authors may wish to confirm the version used and, if applicable, repeat the analysis using the latest version.

Thanks for this comment. We have included a reference to RAAB7 platform (Reference 11) used in this study, which has the most recent version for analysis.

Reviewer #3: Congratulations on a very well and informative paper. I agree that the ECAT is very labor intensive and in many ways, impractical to collect data in some countries.

It would be great to put a link to a reference to further understand the RAAB Health System module and explain if it has already been used elsewhere and how it worked. I am not aware of another study that has used it, but I might be mistaken, and if this is the case what lessons were learned and the limitations that were found, so that future researcher can make an informed decision if they want to use it and how.

Thanks for this comment. This was the first pilot test of this module, and more pilots in other countries followed this study. A publication describing the tool is pending, so we have not been able to add any reference. We have mentioned in our discussion that this is the first pilot of this module (lines 408-409) and have included some of our learnings in the discussion section (lines 369-371) and lines 397-402).

Reviewer #4: I compliment the authors on a well-researched and written manuscript.

The title and stated intent emphasise the “first implementation of the newly developed RAAB Health System Module,” which is described as comprising three sections, A (policies related to eye health), B (socio-demographic characteristics of the population in the sampling area), and C (facility data, including availability and volume of services for cataract, refractive error, and diabetic retinopathy).

In the current submission, the Authors report results only from Section C, despite stating that data were collected for all three sections. Even if Section A is not presented,

Section B should be included because it provides essential context for interpreting both the RAAB findings and the Section C service landscape (for example, catchment population structure and key socio-demographic determinants of service demand and access). The Authors should either incorporate Section B results in the main manuscript, ideally as a concise table aligned to the module framework, or provide Section B as a detailed supplementary appendix with explicit cross-referencing in the Results and Discussion. If Sections A and B are not reported, the title, abstract, and framing should be revised to state explicitly that the paper reports only the Section C facility module rather than the full Health System Module implementation.

Thanks for this suggestion. We have included the Section B and C as Supplementary material (S2_file.excel)

Section C is central to the manuscript’s novelty, but the specific measures captured are not adequately described. The Authors should provide the Section C instrument as an appendix to facilitate readers or a variable dictionary (definitions, units, time window, data sources, handling of missing data).

Thanks for this suggestion. We have included the entire template as supplementary material (S1_file.excel)

% value in Table 5 are not relevant given such low N. Table 5 may be strengthened by including service availability.

Thanks for this suggestion. We have added service availability to the revised Table 5, as suggested, and further details are included in the S1_table.word. We have retained the percentages as standard practice

The Authors report cataract surgery costs ranging from free MSICS to a median INR 7,000 for phacoemulsification, and spectacles pricing by type. The Authors should define whether “cost” is the patient out-of-pocket price, facility charge, or package cost, whether it includes IOL, medicines, investigations, and follow-up etc

Thanks for this suggestion. This was the package cost for the patient, which included IOLs, medicines, and follow-ups. We have mentioned that in line 281.

The Authors state that the number of patients receiving DR screening or treatment could not be estimated due to lack of data. The DR finding should be explicitly framed as a data-gap rather than a service delivery/utilisation estimate. This is an important & relevant result of the Health System Module and should be emphasized in the Discussion as evidence of inadequate routine DR monitoring, with clear separation between service availability and information systems gap (no utilisation data available).

Thanks for this suggestion. We have added additional rows in Table 5 to differentiate between service availability and data availability. We have highlighted the lack of information on DR as an important result in the discussion section. (Lines 383-385 and 391-393)

The manuscript reports multiple group comparisons (for example, sex and age comparisons for prevalence and coverage indicators). For a cluster sampled RAAB design, the Authors should clarify whether inferential testing was survey-adjusted, including clustering (and any weighting if applied).

Thank you for raising this important point regarding survey-adjusted inference.

The RAAB survey followed a two-stage cluster sampling design with probability proportional to size and fixed cluster size. We acknowledge that the reported p-values were derived from conventional Pearson chi-square tests and therefore do not explicitly account for clustering. To assess the potential impact of clustering on inference, we conducted a sensitivity analysis using the design effect (DEFF = 1.4) assumed in RAAB sample size calculations. Under a first-order approximation, dividing the Pearson chi-square statistic by 1.4 inflates the variance to reflect clustering. For example, a chi-square value corresponding to p = 0.001 (χ² = 10.83, df = 1) would yield an adjusted χ² = 7.73 and p = 0.005. Similarly, a borderline χ² = 3.84 (p = 0.05) would correspond to χ² = 2.74 and p = 0.098 under this approximation.

We recognise that this approach represents a heuristic sensitivity analysis and does not replace formal design-based inference, as the true design effect may vary across outcomes. Nonetheless, this exercise suggests that strongly statistically significant associations are likely to remain significant under plausible variance inflation, whereas findings close to the conventional significance threshold may lose statistical significance after adjustment. For this study all our p-values were very small and <0.001. We understand that the 5% level of significance threshold is merely a convention, and interpretation should focus on the magnitude rather than the level of significance. In the revised manuscript, we have therefore clarified in the Methods section that reported p-values are unadjusted for clustering and have emphasised effect sizes in interpretation.

We have added the following sentence in the Methods section of the revised manuscript (Lines 189-191): “*P-values were calculated using Pearson’s chi-square test applied to the entire sample. These tests did not account for clustering in variance estimation and should therefore be interpreted as unadjusted p-values.”*

Authors use age-sex adjusted but that is vague as adjusted is normally implied in multivariate analysis, whereas weighing / direct standardization is what is used when estimating prevalences.

Thanks for this comment. We agree with the reviewer that standardisation is generally used when estimating prevalence. However, as the terminology “age-sex adjusted” is standard in the RAAB methodology and the RAAB report, we have used it. This is also the case with all previous RAAB articles published in this journal. However, we will bring this up with the RAAB Steering Group.

Operational definitions for CSC, eCSC, REC, and eREC should be stated even if RAAB software defaults were used. This is especially relevant since there are variations in how eCSC and eREC are estimated in different surveys. Or reference to the any studies whose estimation methods were used may be given.-

Thanks for your comment. We have included the operational definitions for CSC, eCSC, REC, and eREC in the Methods section. We have also added the operational definition of relative quality gap (Lines-138-146).

Tables 1-4: Authors should provide denominators

Thanks for your comment

We have added the denominator to Table 1 as the last row.

Table 2 had a denominator in the last row, which was the total number of individuals with blindness or different grades of visual impairment in the sample.

Table 3 contains the ratios of coverages and effective coverages as reported by the RAAB software, so the denominators are not included.

Table 4 has two sets of denominators, which have now been clarified. The first set is the total number of operated eyes in the sample (N). This is the denominator for the number of eyes with ‘good, borderline and poor’ outcomes and accounts for the total number of eyes found to have undergone cataract surgery. The second set of denominators is in the last row of the table - total number with non-good outcomes. These are the denominators for reasons for ‘non-good outcomes’ and account for the total number of eyes with “non-good outcomes’

The WHO ECIM gives a range for Cataract surgical outcomes. It would have been good if authors could present based on those

Thanks for this suggestion. We have now included the numbers for all categories, as per that classification, in Table 4. We have retained the reasons for any outcome that was not in the ‘Good’ category as per WHO ECIM.

Post-operative visual acuity - PVA or PinHole VA may be clarified.-

Thanks for this comment. The postoperative visual acuity was based on the presenting vision, as noted in Table 4 now.

We estimated that approximately 200,000 people have mild VI or worse, with about 100,000 cases caused by untreated cataract. -- How have these numbers been arrived at may be stated in Statistical methods

Thanks for this comment. We have modified the number to 100,000 now and that comes from Table1as a total of individuals aged 50 years and above with blindness, severe, moderate or mild visual impairment. To extrapolate the results of the survey to the population, we used the detailed age-group and gender-wise projections for the entire state in the government report. That report projected 16% for this age group in Uttar Pradesh. <https://nhm.gov.in/New_Updates_2018/Report_Population_Projection_2019.pdf> (We have cited that now-reference 12). Due to a data entry error, the age-gender population group counts were double-counted. Now we have corrected that, and the denominator is 318,902 for people aged 50 and above. This denominator is now mentioned in Table 1. The projections of individuals with blindness or any degree of visual impairment among them are also available in Table 1 and that was 100,459. We have corrected that statement to “approximately 100,000 people aged 50 and above have mild VI or worse, with more than 40,000 cases caused by untreated cataract in the catchment” (Lines 303-304)

Lines 348-350: kindly check phrasing/grammar

Thanks, we have modified that now- “CSC and eCSC at a cut-off of 6/12 were 59·6% and 38%. These closely matched the national estimates of 57·3% (95% CI 53·3-61·2%) and 36·7% (95% CI: 33·6, 39·9), respectively.” (lines 332--333)

I find it good that the study has included PCO as a dedicated cause when assessing causes of e non-good outcomes and the high proportions attributed to PCO and potential easy treatment options available justify its inclusion as an independent entity

It is heartening to note that the authors where able to actually make of the survey and HSM data to take critical decisions and adapt their service delivery to better serve the area.

Thanks for the appreciation

Some references need to be properly formatted: Eg 20, 7, 6, 9, 11

Thanks. We have formatted references 6, 7, 6,7,9, 11, and 19 (which was earlier the 20^th^ reference).

I feel that the linkage between RAAB and HSM data is somewhere lacking. This is driven by the fact that a lot of data was missing. The post-operative VA date was available for only one facility which is the authors own facility. DR data was lacking. Refractive error data is not majorly comparable as age groups are different. The DR domain currently cannot be linked at all. The manuscript indicates that DR screening and treatment services exist at two providers, but utilization data for DR screening or treatment are unavailable from all providers.

To me a key learning seems that while RAAB data was helpfile, the HSM data essentially was a mapping of service availability / delivery in the current setting. Except for potentially estimating CSR, other results can be correlated across RAAB and HSM data.

This interpretation is further contained by lack of information on what exactly is measured in HSM module and how it maps to the health system building blocks-

Thanks for this comment. We have now included the entire HSM data collection template as supplementary information. The information intended to be captured in Section C, along with the epidemiological data, can be very useful for planning service delivery, especially for the three common causes of vision loss - cataract, uncorrected refractive errors, and diabetic retinopathy. We agree with the reviewer that, except for service availability and cataract surgical outputs, much of the data was unavailable. We believe this missing information itself is a major finding of this study, and we have mentioned it in our discussion section (lines 379-385). We have also made recommendations that could improve the availability of missing information (lines 385-396). Our study is the first to pilot this tool alongside RAAB in a particular data set. We believe that more such implementations can highlight the specific need for improved data collection across different regions and support better regional planning of eye care services. We have added this in our conclusion (Lines 431-432)
