# Supplementary material for: Beyond prevalence: A complete catchment eye health system profile using rapid assessment of avoidable blindness, a population-based eye health survey, and the first implementation of the newly developed health system module
Source: PLoS One. 2026 Aug 10;21(8):e0354744. doi: 10.1371/journal.pone.0354744 (PMC13456199; doi:10.1371/journal.pone.0354744)
Supplement: Supplementary file 8 [file pone.0354744.s007.docx]

Additional Editor Comments:

Reviewer Comments
Thank you for the revised manuscript. The authors have attempted to incorporate a Health System Module into the existing RAAB framework, which is a welcome and potentially valuable initiative. However, there remain several concerns regarding the feasibility, interpretation, and practical utility of the proposed module and its findings.

Major Comments
1. Feasibility and Validation of the Health System Module
While integrating a Health System Module into RAAB is an interesting concept, it is unclear whether any pilot testing was conducted independently of the RAAB survey to assess the feasibility, completeness, and reliability of collecting such information.
Based on the data presented in the manuscript, the module appears to provide predominantly cataract surgery output-related information, while data related to diabetic retinopathy (DR) and refractive error services are minimal and incomplete. Therefore, statements such as: “It demonstrates how integrating robust epidemiological data with service capability profiling can reveal gaps that remain unseen when either type of data is considered separately” may be overstated and are not adequately supported by the current findings. The manuscript would benefit from a more balanced interpretation of what the Health System Module was actually able to capture in practice.

Our Response:

Thanks for your comment. As indicated in the title, this is the first implementation of this module, and we did not conduct a pilot. Although the module is intended to collect information on services for cataract, refractive error, and diabetic retinopathy, we agree with the reviewer that the available data were mostly limited to cataract delivery. The gaps in the available data were highlighted during implementation and are also noted in our conclusion section in the earlier submitted version - “In addition, the module can highlight gaps in the data required for regional planning.”

We have revised the statement referred to by the reviewer to read (lines 427-432):

“For cataract surgery volume, the analysis shows that integrating robust epidemiological data with service capability profiling can uncover gaps that remain hidden when either data type is analysed separately. However, due to a lack of data on cataract outcomes, refractive errors and DR, the same conclusion cannot be drawn for the assessment of the quality of cataract surgery delivery and the delivery of services for other eye health conditions.”

We have also reported the challenges in the implementation of the Health System Module in the discussion section now (Lines 446-451):

“Although the Health System Module provides the capacity to collect comprehensive data on the three eye conditions, our study highlights feasibility and operational limitations. Data completeness at the facility level, the feasibility of collecting data from small optical shops, reservations about sharing outcome data from private providers, and the lack of open-source outcome data are some of the challenges highlighted by our study. All these can reduce the programmatic utility of the Health System Module.”

We have also added the following in our conclusion(Line 458-460):

“Additionally, the module can identify gaps in the data required for regional planning. Its usefulness can be increased through improved data collection at the facility level and by improving the feasibility of data sharing.”

2. Recommendations Regarding a National Surgical Outcomes Portal
The authors recommend establishing a national portal where organizations could share cataract surgical outcomes anonymously. However, a similar initiative was attempted more than a decade ago by VISION 2020 India and was not successful.
This experience highlights that the challenge is not merely technological but also behavioural and systemic. While having such information would undoubtedly be valuable, successful implementation requires substantial behavioural change, institutional buy-in, trust, and sustained participation. Therefore, studies evaluating strategies to promote behavioural and system-level adoption may be more important at this stage than simply developing additional reporting modules that may prove difficult to implement consistently.

Our Response:

Thanks for this feedback. We have added this now in our discussion (lines 411-414):

“A few years back, a similar national-level initiative to capture cataract outcomes was attempted ^(27)^ but was unsuccessful. This highlights that the issue is not just technical but also behavioural and systemic. Research into strategies to encourage behavioural change and adoption is required.”

3. Programmatic Utility of the Health System Data

The manuscript does not clearly explain how the collected Health System Module data would directly inform program planning or improve service delivery in the surveyed region. The practical application of these data for district-level planning, referral strengthening, workforce deployment, or service improvement needs to be articulated more clearly.
I would therefore suggest that the authors substantially revise the sections related to the Health System Module, including a more detailed discussion of its feasibility, data completeness, operational limitations, challenges in implementation and potential programmatic utility

Our Response:

Thanks, we have added more details now

The following was included in the Introduction in the submitted version:

“This module collects information on policies related to eye services. It captures services offered by both government and non-government eye care providers for three common causes of vision loss: cataract, uncorrected refractive error, and diabetic retinopathy (DR). Since RAABs are typically conducted at the subnational level, this module provides a quick, user-friendly tool to support eye care planning within a survey area. “

We have added this in the introduction to highlight how it can support district-level planning (lines 78-85):

“While RAAB results can help estimate the burden of eye diseases, data on available services are also needed to plan at the district level. This module enables a relatively quick assessment of service gaps for cataract, refractive error, and DR services. The data to be collected in the module include the outputs and outcomes of cataract surgeries, the number of glasses dispensed, vision with glasses dispensed, the number of individuals screened and treated for DR and the human resources at the respective centres providing services. This can support the planning needed to scale up services, improve the service quality, and plan the workforce.”

And in the discussion section, we have highlighted the feasibility and challenges in implementation influencing the programmatic utility (Lines 446-451):

“Although the Health System Module provides the capacity to collect comprehensive data on the three eye conditions, our study highlights feasibility and operational limitations. Data completeness at the facility level, the feasibility of collecting data from small optical shops, reservations about sharing outcome data from private providers, and the lack of open-source outcome data are some of the challenges highlighted by our study. All these can reduce the programmatic utility of the Health System Module”

Additional Comments
4. Table 5
Table 5 provides very limited information and does not add substantial value to the manuscript in its current form. I would suggest removing the table and instead providing the Health System Module link/reference for interested readers.

Our Response:

Thanks for this feedback

We have removed Table 5 from the manuscript and added it as a supplementary table (S1_Table.word). We have renumbered the other supplementary tables accordingly. Instead of the table, we have added the following information in the Results section(Lines 270-279):

“A total of 14 service providers were found to be eligible, and all were included. These included 10 non-governmental organizations (NGOs), 2 government hospitals, and 2 private for-profit organizations. Permanent cataract services were provided by 10 providers, refraction services by 11, glasses dispensing by 9, and screening and treatment of DR by 2. Data on the number of cataract surgeries were available from 12 providers (86%), but only 1 facility (7%) had pre- and post-operative vision data. Data on the cost of cataract surgery for the patient were available from 9 providers (64%). Data on the number of refractions were available from 7 (50%) providers and on dispensed glasses from 4 providers (29%) that offer permanent or occasional refractive services. Data on DR screening or treatment were unavailable from all providers. Details on data availability are included in S1_Table.word.”

5. Interpretation of Corneal Blindness Findings
The manuscript states:
“Corneal opacities were the second leading cause of blindness…”
followed by a detailed description of the tele-ophthalmology-enabled cornea care system already in place.
However, if such systems are already functioning effectively, the persistently high burden of corneal blindness in the region requires further exploration. The discussion currently focuses primarily on available infrastructure rather considering, analysing and discussing the likely reasons for the high burden of corneal blindness in the region and propose targeted strategies to reduce it.

Our Response:

Thanks for this feedback. We agree that other interventions may be required. We have added that to the discussion (Lines 334-339):

“Although this improves the infrastructure for early intervention for corneal conditions among patients presenting to the health system, more needs to be done to promote prevention and encourage early presentations. In this predominantly rural region ^(7)^, agricultural trauma could be an important cause of corneal infections and blindness. ^(16)^ Targeted activities to prevent agricultural injuries, raise awareness of injury prevention, and emphasise the need to seek care early after an injury should be planned.”

6. Inconsistencies in Reported Numbers
There appear to be inconsistencies between the Results and Discussion sections.
For example, in the Discussion the authors state:
“The backlog of cataract surgeries for eyes with VA less than 6/12 … was approximately 130,000, with about 41,000 individuals needing surgeries in both eyes and roughly 48,000 in one eye…”
However, the numbers reported in the Results section differ. These discrepancies should be carefully reviewed and corrected to ensure consistency throughout the manuscript.

Our Response:

Thanks for this comment.

In the results section, we reported that an estimated 41,165 individuals had blindness or any degree of visual impairment (VA less than 6/12) due to cataract in both eyes. For individuals with poor vision in one eye due to cataract, we reported 41,984 for those with vision less than 6/18 ( not less than 6/12). The estimated number of individuals with vision in one eye less than 6/12 due to cataract was 47,840, and this number was included in Supplementary Table 2 in the original submission but not in the text (Supplementary Table 3 in the now-revised version). We have now included this figure in the text in results section. Thus, the backlog of cataract surgeries with a VA threshold of 6/12 is around 82,000 eyes across 41,000 individuals needing surgery in both eyes, plus an additional around 48,000 eyes belonging to individuals needing surgery in one eye. This totals approximately 130,000 eyes needing surgery at a threshold of 6/12, as mentioned in the discussion section.

The revised text in the result section is (Lines 230-235):

“Overall, out of the estimated 100,459 people with blindness or any degree of VI (including mild VI) (Table 1), 41,165 were attributed to unoperated cataract in both eyes (S3_Table.word). There were projected 23,829 individuals with MSVI or blindness due to cataract in both eyes. An additional 41,984 individuals with cataract in one eye with VA less than 6/18 and 47,840 individuals with VA less than 6/12 were estimated. (S3_Table.word)”

And in the discussion section (Line 358):

The backlog of cataract surgeries for eyes with VA less than 6/12 (the cut-off used by WHO for eCSC) was approximately 130,000, with about 41,000 individuals needing surgery in both eyes (82,000 eyes) and almost 48,000 in one eye ( S3_Table.word).

Overall Recommendation
The concept of integrating a Health System Module into RAAB is important and potentially valuable. However, the current manuscript overstates some conclusions relative to the available data and does not sufficiently address the feasibility, implementation challenges, behavioural aspects, and practical utility of the proposed approach.
Hence, substantial revision is required, particularly in relation to the interpretation and discussion of the Health System Module findings and their implications.

Our Response:

Thanks for this feedback. We agree with the reviewer that we did not highlight the feasibility challenges well.

We have now revised the manuscript as per the suggestions

Also, we have added to the discussion section that this module faces implementation and feasibility challenges. The fact that it addresses only three eye conditions was already reported as a limitation in the previous version. (“One limitation of the health system module is that it is intended to collect information only on cataract, refractive error, and DR”). We had also included the challenges in collecting data from all the optical shops in the previous version (“We also could not include information on refraction and glasses sold from optical shops due to logistical reasons.”)

Now we have added the following to the discussion section (Line 446-451):

“Although the Health System Module provides the capacity to collect comprehensive data on the three eye conditions, our study highlights feasibility and operational limitations. Data completeness at the facility level, the feasibility of collecting data from small optical shops, reservations about sharing outcome data from private providers, and the lack of open-source outcome data are some of the challenges highlighted by our study. All these can reduce the programmatic utility of the Health System Module.”

And the following to the conclusions (Line 458-460):

Additionally, the module can identify gaps in the data required for regional planning. Its usefulness can be increased through improved data collection at the facility level and by improving the feasibility of data sharing

[Reviewer's Responses to Questions]

**Comments to the Author**

1. If the authors have adequately addressed your comments raised in a previous round of review and you feel that this manuscript is now acceptable for publication, you may indicate that here to bypass the “Comments to the Author” section, enter your conflict of interest statement in the “Confidential to Editor” section, and submit your "Accept" recommendation.

Reviewer #2: All comments have been addressed

2. Is the manuscript technically sound, and do the data support the conclusions?

The manuscript must describe a technically sound piece of scientific research with data that supports the conclusions. Experiments must have been conducted rigorously, with appropriate controls, replication, and sample sizes. The conclusions must be drawn appropriately based on the data presented.

Reviewer #2: Yes

3. Has the statistical analysis been performed appropriately and rigorously?

Reviewer #2: Yes

4. Have the authors made all data underlying the findings in their manuscript fully available?

The [PLOS Data policy](http://www.plosone.org/static/policies.action#sharing) requires authors to make all data underlying the findings described in their manuscript fully available without restriction, with rare exception (please refer to the Data Availability Statement in the manuscript PDF file). The data should be provided as part of the manuscript or its supporting information, or deposited to a public repository. For example, in addition to summary statistics, the data points behind means, medians and variance measures should be available. If there are restrictions on publicly sharing data—e.g. participant privacy or use of data from a third party—those must be specified.

Reviewer #2: Yes

5. Is the manuscript presented in an intelligible fashion and written in standard English?

PLOS ONE does not copyedit accepted manuscripts, so the language in submitted articles must be clear, correct, and unambiguous. Any typographical or grammatical errors should be corrected at revision, so please note any specific errors here.

Reviewer #2: Yes

6. Review Comments to the Author

Please use the space provided to explain your answers to the questions above. You may also include additional comments for the author, including concerns about dual publication, research ethics, or publication ethics. (Please upload your review as an attachment if it exceeds 20,000 characters)

Reviewer #2: Great work. All my comments are very well addressed. Reviewed the response given to comments by other reviewers and found they are also addressed well.
