# Supplementary material for: Beyond prevalence: A complete catchment eye health system profile using rapid assessment of avoidable blindness, a population-based eye health survey, and the first implementation of the newly developed health system module
Source: PLoS One. 2026 Aug 10;21(8):e0354744. doi: 10.1371/journal.pone.0354744 (PMC13456199; doi:10.1371/journal.pone.0354744)
Supplement: Peer review history

# Peer review: Decision Letter 0

**DOI:** 10.1371/journal.pone.0354744.r001

**Authors:** Rohit C. Khanna

8 Feb 2026

PONE-D-25-63896

Beyond Prevalence: A Complete Catchment Eye Health System Profile Using Rapid Assessment of Avoidable Blindness, a Population-Based Eye Health Survey,and the First Implementation of the Newly Developed Health System Module

PLOS One

Dear Dr. Sabherwal,

Thank you for submitting your manuscript to PLOS ONE. After careful consideration, we feel that it has merit but does not fully meet PLOS ONE’s publication criteria as it currently stands. Therefore, we invite you to submit a revised version of the manuscript that addresses the points raised during the review process.

While Reviewer 1 has recommended rejection, based on the feedback from the other reviewer, we will provide the authors an opportunity to address all the comments. Overall, my observation is that the only new information presented in this paper pertains to the Health System Module, specifically covering areas related to policies, sociodemographic characteristics of the population, and health facility data. This appears to be largely similar to the WHO ECSAT tool. However, the authors have not presented the complete data related to the Health System Module.

Additionally, it is well established that the prevalence of blindness and visual impairment is directly correlated with the cataract surgical rate (CSR) of a country or district. In this context, it is unclear what additional, novel information is generated from the Health System Module that has practical or programmatic implications. Furthermore, the authors need to clarify how information from the Health System Module can be used longitudinally to monitor trends in blindness and visual impairment in a given region.

Please submit your revised manuscript by Mar 25 2026 11:59PM. If you will need more time than this to complete your revisions, please reply to this message or contact the journal office at plosone@plos.org. When you're ready to submit your revision, log on to https://www.editorialmanager.com/pone/ and select the 'Submissions Needing Revision' folder to locate your manuscript file.

Please include the following items when submitting your revised manuscript:

- A letter that responds to each point raised by the academic editor and reviewer(s). You should upload this letter as a separate file labeled 'Response to Reviewers'.
- A marked-up copy of your manuscript that highlights changes made to the original version. You should upload this as a separate file labeled 'Revised Manuscript with Track Changes'.
- An unmarked version of your revised paper without tracked changes. You should upload this as a separate file labeled 'Manuscript'.

If you would like to make changes to your financial disclosure, please include your updated statement in your cover letter. Guidelines for resubmitting your figure files are available below the reviewer comments at the end of this letter.

If applicable, we recommend that you deposit your laboratory protocols in protocols.io to enhance the reproducibility of your results. Protocols.io assigns your protocol its own identifier (DOI) so that it can be cited independently in the future. For instructions see: https://journals.plos.org/plosone/s/submission-guidelines#loc-laboratory-protocols. Additionally, PLOS ONE offers an option for publishing peer-reviewed Lab Protocol articles, which describe protocols hosted on protocols.io. Read more information on sharing protocols at https://plos.org/protocols?utm_medium=editorial-email&utm_source=authorletters&utm_campaign=protocols.

We look forward to receiving your revised manuscript.

Kind regards,

Rohit C. Khanna, MD, MPH

Academic Editor

PLOS One

Journal Requirements:

When submitting your revision, we need you to address these additional requirements.

1. Please ensure that your manuscript meets PLOS ONE's style requirements, including those for file naming. The PLOS ONE style templates can be found at

https://journals.plos.org/plosone/s/file?id=wjVg/PLOSOne_formatting_sample_main_body.pdf and https://journals.plos.org/plosone/s/file?id=ba62/PLOSOne_formatting_sample_title_authors_affiliations.pdf

2. Please describe in your methods section how capacity to provide consent was determined for the participants in this study. Please also state whether your ethics committee or IRB approved this consent procedure. If you did not assess capacity to consent please briefly outline why this was not necessary in this case.

3. Please include a complete copy of PLOS’ questionnaire on inclusivity in global research in your revised manuscript. Our policy for research in this area aims to improve transparency in the reporting of research performed outside of researchers’ own country or community. The policy applies to researchers who have travelled to a different country to conduct research, research with Indigenous populations or their lands, and research on cultural artefacts. The questionnaire can also be requested at the journal’s discretion for any other submissions, even if these conditions are not met. Please find more information on the policy and a link to download a blank copy of the questionnaire here: https://journals.plos.org/plosone/s/best-practices-in-research-reporting. Please upload a completed version of your questionnaire as Supporting Information when you resubmit your manuscript.

4. Thank you for stating the following in the Acknowledgments Section of your manuscript:

[Co-authors on this project are supported by the National Institute for Health Research (NIHR) (using the UK’s Official Development Assistance (ODA) Funding) and Wellcome [215633/Z/19/Z] under the NIHR-Wellcome Partnership for Global Health Research. The views expressed are those of the authors and not necessarily those of Wellcome, the NIHR or the Department of Health and Social Care]

We note that you have provided funding information that is not currently declared in your Funding Statement. However, funding information should not appear in the Acknowledgments section or other areas of your manuscript. We will only publish funding information present in the Funding Statement section of the online submission form.

Please remove any funding-related text from the manuscript and let us know how you would like to update your Funding Statement. Currently, your Funding Statement reads as follows:

[The author(s) received no specific funding for this work.]

Please include your amended statements within your cover letter; we will change the online submission form on your behalf.

5. Please note that your Data Availability Statement is currently missing the repository name and/or the DOI/accession number of each dataset. If your manuscript is accepted for publication, you will be asked to provide these details on a very short timeline. We therefore suggest that you provide this information now, though we will not hold up the peer review process if you are unable.

6. Your ethics statement should only appear in the Methods section of your manuscript. If your ethics statement is written in any section besides the Methods, please delete it from any other section.

7. Please remove your figures from within your manuscript file, leaving only the individual TIFF/EPS image files, uploaded separately. These will be automatically included in the reviewers’ PDF.

8. We note that Figure 1 in your submission contains map images which may be copyrighted. All PLOS content is published under the Creative Commons Attribution License (CC BY 4.0), which means that the manuscript, images, and Supporting Information files will be freely available online, and any third party is permitted to access, download, copy, distribute, and use these materials in any way, even commercially, with proper attribution. For these reasons, we cannot publish previously copyrighted maps or satellite images created using proprietary data, such as Google software (Google Maps, Street View, and Earth). For more information, see our copyright guidelines: http://journals.plos.org/plosone/s/licenses-and-copyright.

We require you to either (1) present written permission from the copyright holder to publish these figures specifically under the CC BY 4.0 license, or (2) remove the figures from your submission:

1. You may seek permission from the original copyright holder of Figure 1 to publish the content specifically under the CC BY 4.0 license.

We recommend that you contact the original copyright holder with the Content Permission Form (http://journals.plos.org/plosone/s/file?id=7c09/content-permission-form.pdf) and the following text:

“I request permission for the open-access journal PLOS ONE to publish XXX under the Creative Commons Attribution License (CCAL) CC BY 4.0 (http://creativecommons.org/licenses/by/4.0/). Please be aware that this license allows unrestricted use and distribution, even commercially, by third parties. Please reply and provide explicit written permission to publish XXX under a CC BY license and complete the attached form.”

Please upload the completed Content Permission Form or other proof of granted permissions as an "Other" file with your submission.

In the figure caption of the copyrighted figure, please include the following text: “Reprinted from [ref] under a CC BY license, with permission from [name of publisher], original copyright [original copyright year].”

2. If you are unable to obtain permission from the original copyright holder to publish these figures under the CC BY 4.0 license or if the copyright holder’s requirements are incompatible with the CC BY 4.0 license, please either i) remove the figure or ii) supply a replacement figure that complies with the CC BY 4.0 license. Please check copyright information on all replacement figures and update the figure caption with source information. If applicable, please specify in the figure caption text when a figure is similar but not identical to the original image and is therefore for illustrative purposes only.

The following resources for replacing copyrighted map figures may be helpful:

USGS National Map Viewer (public domain): http://viewer.nationalmap.gov/viewer/

The Gateway to Astronaut Photography of Earth (public domain): http://eol.jsc.nasa.gov/sseop/clickmap/

Maps at the CIA (public domain): https://www.cia.gov/library/publications/the-world-factbook/index.html and https://www.cia.gov/library/publications/cia-maps-publications/index.html

NASA Earth Observatory (public domain): http://earthobservatory.nasa.gov/

Landsat: http://landsat.visibleearth.nasa.gov/

USGS EROS (Earth Resources Observatory and Science (EROS) Center) (public domain): http://eros.usgs.gov/#

Natural Earth (public domain): http://www.naturalearthdata.com/

9. Please include captions for your Supporting Information files at the end of your manuscript, and update any in-text citations to match accordingly. Please see our Supporting Information guidelines for more information: http://journals.plos.org/plosone/s/supporting-information.

If the reviewer comments include a recommendation to cite specific previously published works, please review and evaluate these publications to determine whether they are relevant and should be cited. There is no requirement to cite these works unless the editor has indicated otherwise.

Additional Editor Comments:

While Reviewer 1 has recommended rejection, based on the feedback from the other reviewer, we will provide the authors an opportunity to address all the comments. Overall, my observation is that the only new information presented in this paper pertains to the Health System Module, specifically covering areas related to policies, sociodemographic characteristics of the population, and health facility data. This appears to be largely similar to the WHO ECSAT tool. However, the authors have not presented the complete data related to the Health System Module.

Additionally, it is well established that the prevalence of blindness and visual impairment is directly correlated with the cataract surgical rate (CSR) of a country or district. In this context, it is unclear what additional, novel information is generated from the Health System Module that has practical or programmatic implications. Furthermore, the authors need to clarify how information from the Health System Module can be used longitudinally to monitor trends in blindness and visual impairment in a given region.

[Note: HTML markup is below. Please do not edit.]

Reviewers' comments:

Reviewer's Responses to Questions

**Comments to the Author**

1. Is the manuscript technically sound, and do the data support the conclusions?

The manuscript must describe a technically sound piece of scientific research with data that supports the conclusions. Experiments must have been conducted rigorously, with appropriate controls, replication, and sample sizes. The conclusions must be drawn appropriately based on the data presented.

Reviewer #1: No

Reviewer #2: Yes

Reviewer #3: Yes

Reviewer #4: Yes

**********

2. Has the statistical analysis been performed appropriately and rigorously?

Reviewer #1: N/A

Reviewer #2: Yes

Reviewer #3: Yes

Reviewer #4: Yes

**********

3. Have the authors made all data underlying the findings in their manuscript fully available?

The PLOS Data policy requires authors to make all data underlying the findings described in their manuscript fully available without restriction, with rare exception (please refer to the Data Availability Statement in the manuscript PDF file). The data should be provided as part of the manuscript or its supporting information, or deposited to a public repository. For example, in addition to summary statistics, the data points behind means, medians and variance measures should be available. If there are restrictions on publicly sharing data—e.g. participant privacy or use of data from a third party—those must be specified.

Reviewer #1: Yes

Reviewer #2: Yes

Reviewer #3: Yes

Reviewer #4: Yes

**********

4. Is the manuscript presented in an intelligible fashion and written in standard English?

PLOS ONE does not copyedit accepted manuscripts, so the language in submitted articles must be clear, correct, and unambiguous. Any typographical or grammatical errors should be corrected at revision, so please note any specific errors here.

Reviewer #1: No

Reviewer #2: Yes

Reviewer #3: Yes

Reviewer #4: Yes

**********

5. Review Comments to the Author

Please use the space provided to explain your answers to the questions above. You may also include additional comments for the author, including concerns about dual publication, research ethics, or publication ethics. (Please upload your review as an attachment if it exceeds 20,000 characters)

Reviewer #1: I appreciate the hard work put into amalgamating the findings of the RAAB survey and the limited analysis of the situation of eye care services in the study area.

The manuscript is well written. However, it has limited value for planning eye health care programs at the primary, secondary, and tertiary levels and covers preventive, curative, and rehabilitative components of eye health. It may send wrong messages to planners of developing countries to blindly use the outcome of the present study to strengthen eye services.

Reviewer #2: Congratulations to the authors for preparing a manuscript that is highly relevant for eye health planning in the region. The integration of the health system module into RAAB adds significant value by enabling an assessment of current service capacity, which can serve as an important input for effective eye care planning.

Overall, the manuscript is well written. The use of well-established study protocols and the high response rate reflect the systematic execution of the survey. The results are clearly presented based on outputs generated from the RAAB software, and the manuscript covers the major indicators related to cataract services and distance refractive error.

However, I have the following queries and requests for clarification:

1. Line 268: The proportion of individuals with good outcomes was significantly higher among those operated on within two years of the survey compared to those operated on earlier (74.4% vs 57.0%; p < 0.0001).

It would be helpful if the authors could discuss potential factors underlying this difference in visual acuity outcomes, such as changes in refractive error over time or the manifestation of other ocular comorbidities.

2. Table 1: Please clarify how the figure 208,929 was derived. This number does not appear to align with the population details provided. Kindly specify the denominator used to arrive at this estimate.

3. Table 3: The reported distance effective refractive error coverage (eREC) appears to be considerably lower compared to a recent publication in the AJOI based on a study conducted in Theni district, which reported more than double this rate. The authors may consider including this reference and briefly discussing possible reasons for the observed difference.

4. Line 237: Please clarify how the figure 85,394 was calculated.

The overall population of the study region is reported as 2,034,568, with individuals aged ≥50 years constituting 20% of the population estimated as 406,974. However, most of the rates are relating to a population of 656,000+. Additionally, as the rates presented are based on the primary cause of blindness, the actual cataract burden may be higher than the proportions reported in Table 2. The authors may wish to clarify or comment on this aspect.

Given that RAAB has been updated regularly and that some analytical issues have been resolved in newer versions, the authors may wish to confirm the version used and, if applicable, repeat the analysis using the latest version.

Reviewer #3: Congratulations on a very well and informative paper. I agree that the ECAT is very labor intensive and in many ways, impractical to collect data in some countries. It would be great to put a link to a reference to further understand the RAAB Health System module and explain if it has already been used elsewhere and how it worked. I am not aware of another study that has used it, but I might be mistaken, and if this is the case what lessons were learned and the limitations that were found, so that future researcher can make an informed decision if they want to use it and how.

Reviewer #4: I compliment the authors on a well-researched and written manuscript.

The title and stated intent emphasise the “first implementation of the newly developed RAAB Health System Module,” which is described as comprising three sections, A (policies related to eye health), B (socio-demographic characteristics of the population in the sampling area), and C (facility data, including availability and volume of services for cataract, refractive error, and diabetic retinopathy).

In the current submission, the Authors report results only from Section C, despite stating that data were collected for all three sections. Even if Section A is not presented, Section B should be included because it provides essential context for interpreting both the RAAB findings and the Section C service landscape (for example, catchment population structure and key socio-demographic determinants of service demand and access). The Authors should either incorporate Section B results in the main manuscript, ideally as a concise table aligned to the module framework, or provide Section B as a detailed supplementary appendix with explicit cross-referencing in the Results and Discussion. If Sections A and B are not reported, the title, abstract, and framing should be revised to state explicitly that the paper reports only the Section C facility module rather than the full Health System Module implementation.

Section C is central to the manuscript’s novelty, but the specific measures captured are not adequately described. The Authors should provide the Section C instrument as an appendix to facilitate readers or a variable dictionary (definitions, units, time window, data sources, handling of missing data).

% value in Table 5 are not relevant given such low N. Table 5 May be strengthened by including service availability.

The Authors report cataract surgery costs ranging from free MSICS to a median INR 7,000 for phacoemulsification, and spectacles pricing by type. The Authors should define whether “cost” is the patient out-of-pocket price, facility charge, or package cost, whether it includes IOL, medicines, investigations, and follow-up etc

The Authors state that the number of patients receiving DR screening or treatment could not be estimated due to lack of data. The DR finding should be explicitly framed as a data-gap rather than a service delivery/utilisation estimate. This is an important & relevant result of the Health System Module and should be emphasized in the Discussion as evidence of inadequate routine DR monitoring, with clear separation between service availability and information systems gap (no utilisation data available).

The manuscript reports multiple group comparisons (for example, sex and age comparisons for prevalence and coverage indicators). For a cluster sampled RAAB design, the Authors should clarify whether inferential testing was survey-adjusted, including clustering (and any weighting if applied). Authors use age-sex adjusted but that is vague as adjusted is normally implied in multivariate analysis, whereas weighing / direct standardization is what is used when estimating prevalences.

Operational definitions for CSC, eCSC, REC, and eREC should be stated even if RAAB software defaults were used. This is especially relevant since there are variations in how eCSC and eREC are estimated in different surveys. Or reference to the any studies whose estimation methods were used may be given.

Tables 1-4: Authors should provide denominators in Table header rows

The WHO ECIM gives a range for Cataract surgical outcomes. It would have been good if authors could present based on those.

Post-operative visual acuity - PVA or PinHole VA may be clarified.

We estimated that approximately 200,000 people have mild VI or worse, with about 100,000 cases caused by untreated cataract. -- How have these numbers been arrived at may be stated in Statistical methods

Lines 348-350: kindly check phrasing/grammar

I find it good that the study has included PCO as a dedicated cause when assessing causes of e non-good outcomes and the high proportions attributed to PCO and p[otential easy treatment options available justify its inclusion as an independent entity

It is heartening to note that the authors where able to actually make of the survey and HSM data to take critical decisions and adapt their service delivery to better serve the area.

Some references need to be properly formatted: Eg 20, 7, 6, 9, 11

I feel that the linkage between RAAB and HSM data is somewhere lacking. This is driven by the fact that a lot of data was missing. The post-operative VA date was available for only one facility which is the authors own facility. DR data was lacking. Refractive error data is not majorly comparable as age groups are different. The DR domain currently cannot be linked at all. The manuscript indicates that DR screening and treatment services exist at two providers, but utilization data for DR screening or treatment are unavailable from all providers.

To me a key learning seems that while RAAB data was helpfile, the HSM data essentially was a mapping of service availability / delivery in the current setting. Except for potentially estimating CSR, other results can be correlated across RAAB and HSM data.

This interpretation is further contained by lack of information on what exactly is measured in HSM module and how it maps to the health system building blocks

**********

6. PLOS authors have the option to publish the peer review history of their article (what does this mean?). If published, this will include your full peer review and any attached files.

If you choose “no”, your identity will remain anonymous but your review may still be made public.

**Do you want your identity to be public for this peer review?**  For information about this choice, including consent withdrawal, please see our Privacy Policy.

Reviewer #1: No

Reviewer #2: **Yes:**  Ganesh Babu Balu Subburaman

Reviewer #3: No

Reviewer #4: **Yes:**  Dr Vivek Gupta

**********

[NOTE: If reviewer comments were submitted as an attachment file, they will be attached to this email and accessible via the submission site. Please log into your account, locate the manuscript record, and check for the action link "View Attachments". If this link does not appear, there are no attachment files.]

To ensure your figures meet our technical requirements, please review our figure guidelines: https://journals.plos.org/plosone/s/figures

You may also use PLOS’s free figure tool, NAAS, to help you prepare publication quality figures: https://journals.plos.org/plosone/s/figures#loc-tools-for-figure-preparation.

NAAS will assess whether your figures meet our technical requirements by comparing each figure against our figure specifications.

# Author comment: Author response to Decision Letter 1

**DOI:** 10.1371/journal.pone.0354744.r002

9 Mar 2026

Our responses are shown in blue font in the document attached, and the page numbers refer to the revised clean copy.

While Reviewer 1 has recommended rejection, based on the feedback from the other reviewer, we will provide the authors an opportunity to address all the comments. Overall, my observation is that the only new information presented in this paper pertains to the Health System Module, specifically covering areas related to policies, sociodemographic characteristics of the population, and health facility data. This appears to be largely similar to the WHO ECSAT tool. However, the authors have not presented the complete data related to the Health System Module.

Response: Thanks for your comments and this opportunity. In the manuscript, we have included the results from the section with health facility data. However, we have now added the results of the other two sections as supplementary files. Although this tool is similar to the ECSAT tool, this can be deployed more rapidly and is less resource-intensive. WHO ECSAT is generally deployed at the national level, whereas this tool is intended for the sub-national level. As RAABs are generally implemented at the sub-national level, this tool could supplement RAAB data for planning services in the region of the RAAB survey. We have highlighted this difference in the ‘Discussion section (lines 298-302)

Additionally, it is well established that the prevalence of blindness and visual impairment is directly correlated with the cataract surgical rate (CSR) of a country or district. In this context, it is unclear what additional, novel information is generated from the Health System Module that has practical or programmatic implications.

Response: Thanks for your comment. Information generated by the Health System Module includes the availability of existing eye care services in the sub-national region under study. This tool is not intended to comment on the prevalence of blindness or visual impairment. It provides information on the prevailing eye care services at the facility level in the region, and this information can supplement the prevalence data collected from surveys like RAAB to plan services based on needs and gaps. Thus, we believe this has programmatic implications.

In our study, it was shown that, despite the lead author’s facility being the primary service provider, other providers were involved. Thus, data from all such facilities, along with data on need in this region from the RAAB study, were used for planning services. (Lines 343-348). In lines 408-411, we mentioned that this model helped estimate the service gap using prevalence and facility data.

Furthermore, the authors need to clarify how information from the Health System Module can be used longitudinally to monitor trends in blindness and visual impairment in a given region.

Response: Information from Health System Module is not related to the prevalence of blindness and visual impairment, and thus trends in blindness and visual impairment cannot be monitored using this. However, as Health System Module can be repeated every few years, trends in overall service provision in the region can be estimated.

Please describe in your methods section how capacity to provide consent was determined for the participants in this study. Please also state whether your ethics committee or IRB approved this consent procedure. If you did not assess capacity to consent please briefly outline why this was not necessary in this case.

Response: Thanks for your comment. We have added the exclusion criteria that were approved by our ethics committee and followed (Line 100-104)

“Individuals aged 50 and above who had been ordinarily resident for at least 6 months prior to the survey and who consented were included in the study. Visitors and those unwilling to consent were excluded. In addition, those found to be mentally unsound and unable to provide consent were excluded. This was assessed by the ophthalmologist, who was the clinical lead for each team. This assessment was based on the coherence of responses during the general introductory conversation and the ability to communicate.”

3. Please include a complete copy of PLOS’ questionnaire on inclusivity in global research in your revised manuscript. Our policy for research in this area aims to improve transparency in the reporting of research performed outside of researchers’ own country or community. The policy applies to researchers who have travelled to a different country to conduct research, research with Indigenous populations or their lands, and research on cultural artefacts. The questionnaire can also be requested at the journal’s discretion for any other submissions, even if these conditions are not met. Please find more information on the policy and a link to download a blank copy of the questionnaire here: https://journals.plos.org/plosone/s/best-practices-in-research-reporting. Please upload a completed version of your questionnaire as Supporting Information when you resubmit your manuscript.

Response: Filled and uploaded

4. Thank you for stating the following in the Acknowledgments Section of your manuscript:

[Co-authors on this project are supported by the National Institute for Health Research (NIHR) (using the UK’s Official Development Assistance (ODA) Funding) and Wellcome [215633/Z/19/Z] under the NIHR-Wellcome Partnership for Global Health Research. The views expressed are those of the authors and not necessarily those of Wellcome, the NIHR or the Department of Health and Social Care]

We note that you have provided funding information that is not currently declared in your Funding Statement. However, funding information should not appear in the Acknowledgements section or other areas of your manuscript. We will only publish funding information present in the Funding Statement section of the online submission form.

Please remove any funding-related text from the manuscript and let us know how you would like to update your Funding Statement. Currently, your Funding Statement reads as follows:

[The author(s) received no specific funding for this work.]

Please include your amended statements within your cover letter; we will change the online submission form on your behalf.

Response: No specific funding for this research was received from the sources mentioned in the acknowledgement. We had included those statements because some of the co-authors were supported by the grants mentioned. We would like to seek editorial approval to include those statement and have removed that from the manuscript.

As no specific funding was received by the authors for this study, our Funding Statement remains the same

5. Please note that your Data Availability Statement is currently missing the repository name and/or the DOI/accession number of each dataset. If your manuscript is accepted for publication, you will be asked to provide these details on a very short timeline. We therefore suggest that you provide this information now, though we will not hold up the peer review process if you are unable.

Response: We have included that now (lines 536)

https://www.doi.org/10.17037/RAABDATASET.b871319b-08ee-4508-bd41-413733c51075

6. Your ethics statement should only appear in the Methods section of your manuscript. If your ethics statement is written in any section besides the Methods, please delete it from any other section.

Response: We have done this now

7. Please remove your figures from within your manuscript file, leaving only the individual TIFF/EPS image files, uploaded separately. These will be automatically included in the reviewers’ PDF.

Response: We have removed the figure now

8. We note that Figure 1 in your submission contains map images which may be copyrighted. All PLOS content is published under the Creative Commons Attribution License (CC BY 4.0), which means that the manuscript, images, and Supporting Information files will be freely available online, and any third party is permitted to access, download, copy, distribute, and use these materials in any way, even commercially, with proper attribution. For these reasons, we cannot publish previously copyrighted maps or satellite images created using proprietary data, such as Google software (Google Maps, Street View, and Earth). For more information, see our copyright guidelines: http://journals.plos.org/plosone/s/licenses-and-copyright.

We require you to either (1) present written permission from the copyright holder to publish these figures specifically under the CC BY 4.0 license, or (2) remove the figures from your submission:

1. You may seek permission from the original copyright holder of Figure 1 to publish the content specifically under the CC BY 4.0 license.

We recommend that you contact the original copyright holder with the Content Permission Form (http://journals.plos.org/plosone/s/file?id=7c09/content-permission-form.pdf) and the following text:

“I request permission for the open-access journal PLOS ONE to publish XXX under the Creative Commons Attribution License (CCAL) CC BY 4.0 (http://creativecommons.org/licenses/by/4.0/). Please be aware that this license allows unrestricted use and distribution, even commercially, by third parties. Please reply and provide explicit written permission to publish XXX under a CC BY license and complete the attached form.”

Please upload the completed Content Permission Form or other proof of granted permissions as an "Other" file with your submission.

In the figure caption of the copyrighted figure, please include the following text: “Reprinted from [ref] under a CC BY license, with permission from [name of publisher], original copyright [original copyright year].”

2. If you are unable to obtain permission from the original copyright holder to publish these figures under the CC BY 4.0 license or if the copyright holder’s requirements are incompatible with the CC BY 4.0 license, please either i) remove the figure or ii) supply a replacement figure that complies with the CC BY 4.0 license. Please check copyright information on all replacement figures and update the figure caption with source information. If applicable, please specify in the figure caption text when a figure is similar but not identical to the original image and is therefore for illustrative purposes only.

The following resources for replacing copyrighted map figures may be helpful:

USGS National Map Viewer (public domain): http://viewer.nationalmap.gov/viewer/

The Gateway to Astronaut Photography of Earth (public domain): http://eol.jsc.nasa.gov/sseop/clickmap/

Maps at the CIA (public domain): https://www.cia.gov/library/publications/the-world-factbook/index.html and https://www.cia.gov/library/publications/cia-maps-publications/index.html

NASA Earth Observatory (public domain): http://earthobservatory.nasa.gov/

Landsat: http://landsat.visibleearth.nasa.gov/

USGS EROS (Earth Resources Observatory and Science (EROS) Center) (public domain): http://eros.usgs.gov/#

Natural Earth (public domain): http://www.naturalearthdata.com/

Response:Thanks for your feedback. For the revised figure, we have sourced maps from the Natural Earth (public domain) website (https://www.naturalearthdata.com/downloads/50m-cultural-vectors/50m-admin-1-states-provinces/) and then modified them.

9. Please include captions for your Supporting Information files at the end of your manuscript, and update any in-text citations to match accordingly. Please see our Supporting Information guidelines for more – see how to add caption

information: http://journals.plos.org/plosone/s/supporting-information.

Response: Thanks, we have done this now (Lines 577-581)

If the reviewer comments include a recommendation to cite specific previously published works, please review and evaluate these publications to determine whether they are relevant and should be cited. There is no requirement to cite these works unless the editor has indicated otherwise.

5. Review Comments to the Author

Please use the space provided to explain your answers to the questions above. You may also include additional comments for the author, including concerns about dual publication, research ethics, or publication ethics. (Please upload your review as an attachment if it exceeds 20,000 characters)

Reviewer #1: I appreciate the hard work put into amalgamating the findings of the RAAB survey and the limited analysis of the situation of eye care services in the study area.

The manuscript is well written. However, it has limited value for planning eye health care programs at the primary, secondary, and tertiary levels and covers preventive, curative, and rehabilitative components of eye health. It may send wrong messages to planners of developing countries to blindly use the outcome of the present study to strengthen eye services.

Resonse: Thanks for your comments. Based on global eye health stakeholder feedback (pending publication), this health system module prioritises three common causes of vision loss: cataract, uncorrected refractive error, and diabetic retinopathy. We also believe that restricting the module to common conditions also makes it relatively rapid and less resource-intensive.

We believe that the information in this module can effectively supplement epidemiological information obtained from RAAB, which is also generally used for planning purposes. However, we have acknowledged as a limitation in the discussion section that only three common eye conditions are included in this module. (lines 403-407)

Reviewer #2: Congratulations to the authors for preparing a manuscript that is highly relevant for eye health planning in the region. The integration of the health system module into RAAB adds significant value by enabling an assessment of current service capacity, which can serve as an important input for effective eye care planning.

Overall, the manuscript is well written. The use of well-established study protocols and the high response rate reflect the systematic execution of the survey. The results are clearly presented based on outputs generated from the RAAB software, and the manuscript covers the major indicators related to cataract services and distance refractive error.

However, I have the following queries and requests for clarification:

1. Line 268: The proportion of individuals with good outcomes was significantly higher among those operated on within two years of the survey compared to those operated on earlier (74.4% vs 57.0%; p < 0.0001).

It would be helpful if the authors could discuss potential factors underlying this difference in visual acuity outcomes, such as changes in refractive error over time or the manifestation of other ocular comorbidities.

Response: Thanks for this comments, for bringing this out. We have added some potential factors now (line 314-318)

“This trend towards better outcomes could be due to improved infrastructure, better-trained human resources, including surgeons, and a higher proportion of patients undergoing phacoemulsification with better uncorrected visual acuity. However, collecting cataract surgical facility data on trends in these factors was beyond the scope of this study.”

2. Table 1: Please clarify how the figure 208,929 was derived. This number does not appear to align with the population details provided. Kindly specify the denominator used to arrive at this estimate.

Response: Thank you for identifying and highlighting this.

Following the completion of the study, we used the age distribution estimates as per the government projection report on population. (16%) https://nhm.gov.in/New_Updates_2018/Report_Population_Projection_2019.pdf,

for extrapolation to the population. (We have cited that now-reference 12). Due to a data entry error, the age-gender population group counts were double-counted. We have now corrected that, and the estimated number of individuals aged 50 and above is 318,902 (the new denominator for extrapolating the prevalence of blindness and visual impairment from the results of RAAB in that population; the denominator is now included in the last row of Table 1). Using that as the new denominator, the number of individuals with blindness or any degree of visual impairment (

Attachment Submitted filename: RAAB HS_Plos one_response to reviewers comments_Fig.docx

# Peer review: Decision Letter 1

**DOI:** 10.1371/journal.pone.0354744.r003

**Authors:** Rohit C. Khanna

14 Jun 2026

<div>PONE-D-25-63896R1

Beyond Prevalence: A Complete Catchment Eye Health System Profile Using Rapid Assessment of Avoidable Blindness, a Population-Based Eye Health Survey,and the First Implementation of the Newly Developed Health System Module

PLOS One

Dear Dr. Sabherwal

Thank you for submitting your manuscript to PLOS ONE. After careful consideration, we feel that it has merit but does not fully meet PLOS ONE’s publication criteria as it currently stands. Therefore, we invite you to submit a revised version of the manuscript that addresses the points raised during the review process.

Please submit your revised manuscript by Jul 29 2026 11:59PM. If you will need more time than this to complete your revisions, please reply to this message or contact the journal office at plosone@plos.org. When you're ready to submit your revision, log on to https://www.editorialmanager.com/pone/ and select the 'Submissions Needing Revision' folder to locate your manuscript file.

Please include the following items when submitting your revised manuscript:

- A letter that responds to each point raised by the academic editor and reviewer(s). You should upload this letter as a separate file labeled 'Response to Reviewers'.
- A marked-up copy of your manuscript that highlights changes made to the original version. You should upload this as a separate file labeled 'Revised Manuscript with Track Changes'.
- An unmarked version of your revised paper without tracked changes. You should upload this as a separate file labeled 'Manuscript'.

If you would like to make changes to your financial disclosure, please include your updated statement in your cover letter. Guidelines for resubmitting your figure files are available below the reviewer comments at the end of this letter.

If applicable, we recommend that you deposit your laboratory protocols in protocols.io to enhance the reproducibility of your results. Protocols.io assigns your protocol its own identifier (DOI) so that it can be cited independently in the future. For instructions see: https://journals.plos.org/plosone/s/submission-guidelines#loc-laboratory-protocols. Additionally, PLOS ONE offers an option for publishing peer-reviewed Lab Protocol articles, which describe protocols hosted on protocols.io. Read more information on sharing protocols at https://plos.org/protocols?utm_medium=editorial-email&utm_source=authorletters&utm_campaign=protocols.

As the corresponding author, your ORCID iD is verified in the submission system and will appear in the published article. PLOS supports the use of ORCID, and we encourage all coauthors to register for an ORCID iD and use it as well. Please encourage your coauthors to verify their ORCID iD within the submission system before final acceptance, as unverified ORCID iDs will not appear in the published article. *Only*  the individual author can complete the verification step; PLOS staff *cannot*  verify ORCID iDs on behalf of authors.

We look forward to receiving your revised manuscript.

Kind regards,

Rohit C. Khanna, MD, MPH

Academic Editor

PLOS One

Journal Requirements:

If the reviewer comments include a recommendation to cite specific previously published works, please review and evaluate these publications to determine whether they are relevant and should be cited. There is no requirement to cite these works unless the editor has indicated otherwise.

Additional Editor Comments:

Reviewer Comments

Thank you for the revised manuscript. The authors have attempted to incorporate a Health System Module into the existing RAAB framework, which is a welcome and potentially valuable initiative. However, there remain several concerns regarding the feasibility, interpretation, and practical utility of the proposed module and its findings.

Major Comments

1. Feasibility and Validation of the Health System Module

While integrating a Health System Module into RAAB is an interesting concept, it is unclear whether any pilot testing was conducted independently of the RAAB survey to assess the feasibility, completeness, and reliability of collecting such information.

Based on the data presented in the manuscript, the module appears to provide predominantly cataract surgery output-related information, while data related to diabetic retinopathy (DR) and refractive error services are minimal and incomplete. Therefore, statements such as: “It demonstrates how integrating robust epidemiological data with service capability profiling can reveal gaps that remain unseen when either type of data is considered separately” may be overstated and are not adequately supported by the current findings. The manuscript would benefit from a more balanced interpretation of what the Health System Module was actually able to capture in practice.

2. Recommendations Regarding a National Surgical Outcomes Portal

The authors recommend establishing a national portal where organizations could share cataract surgical outcomes anonymously. However, a similar initiative was attempted more than a decade ago by VISION 2020 India and was not successful.

This experience highlights that the challenge is not merely technological but also behavioural and systemic. While having such information would undoubtedly be valuable, successful implementation requires substantial behavioural change, institutional buy-in, trust, and sustained participation. Therefore, studies evaluating strategies to promote behavioural and system-level adoption may be more important at this stage than simply developing additional reporting modules that may prove difficult to implement consistently.

3. Programmatic Utility of the Health System Data

The manuscript does not clearly explain how the collected Health System Module data would directly inform program planning or improve service delivery in the surveyed region. The practical application of these data for district-level planning, referral strengthening, workforce deployment, or service improvement needs to be articulated more clearly.

I would therefore suggest that the authors substantially revise the sections related to the Health System Module, including a more detailed discussion of its feasibility, data completeness, operational limitations, challenges in implementation and potential programmatic utility

Additional Comments

4. Table 5

Table 5 provides very limited information and does not add substantial value to the manuscript in its current form. I would suggest removing the table and instead providing the Health System Module link/reference for interested readers.

5. Interpretation of Corneal Blindness Findings

The manuscript states:

“Corneal opacities were the second leading cause of blindness…”

followed by a detailed description of the tele-ophthalmology-enabled cornea care system already in place.

However, if such systems are already functioning effectively, the persistently high burden of corneal blindness in the region requires further exploration. The discussion currently focuses primarily on available infrastructure rather considering, analysing and discussing the likely reasons for the high burden of corneal blindness in the region and propose targeted strategies to reduce it.

6. Inconsistencies in Reported Numbers

There appear to be inconsistencies between the Results and Discussion sections.

For example, in the Discussion the authors state:

“The backlog of cataract surgeries for eyes with VA less than 6/12 … was approximately 130,000, with about 41,000 individuals needing surgeries in both eyes and roughly 48,000 in one eye…”

However, the numbers reported in the Results section differ. These discrepancies should be carefully reviewed and corrected to ensure consistency throughout the manuscript.

Overall Recommendation

The concept of integrating a Health System Module into RAAB is important and potentially valuable. However, the current manuscript overstates some conclusions relative to the available data and does not sufficiently address the feasibility, implementation challenges, behavioural aspects, and practical utility of the proposed approach.

Hence, substantial revision is required, particularly in relation to the interpretation and discussion of the Health System Module findings and their implications.

[Note: HTML markup is below. Please do not edit.]

Reviewers' comments:

Reviewer's Responses to Questions

**Comments to the Author**

1. If the authors have adequately addressed your comments raised in a previous round of review and you feel that this manuscript is now acceptable for publication, you may indicate that here to bypass the “Comments to the Author” section, enter your conflict of interest statement in the “Confidential to Editor” section, and submit your "Accept" recommendation.

Reviewer #2: All comments have been addressed

**********

2. Is the manuscript technically sound, and do the data support the conclusions?

The manuscript must describe a technically sound piece of scientific research with data that supports the conclusions. Experiments must have been conducted rigorously, with appropriate controls, replication, and sample sizes. The conclusions must be drawn appropriately based on the data presented.

Reviewer #2: Yes

**********

3. Has the statistical analysis been performed appropriately and rigorously?

Reviewer #2: Yes

**********

4. Have the authors made all data underlying the findings in their manuscript fully available?

The PLOS Data policy requires authors to make all data underlying the findings described in their manuscript fully available without restriction, with rare exception (please refer to the Data Availability Statement in the manuscript PDF file). The data should be provided as part of the manuscript or its supporting information, or deposited to a public repository. For example, in addition to summary statistics, the data points behind means, medians and variance measures should be available. If there are restrictions on publicly sharing data—e.g. participant privacy or use of data from a third party—those must be specified.

Reviewer #2: Yes

**********

5. Is the manuscript presented in an intelligible fashion and written in standard English?

PLOS ONE does not copyedit accepted manuscripts, so the language in submitted articles must be clear, correct, and unambiguous. Any typographical or grammatical errors should be corrected at revision, so please note any specific errors here.

Reviewer #2: Yes

**********

6. Review Comments to the Author

Please use the space provided to explain your answers to the questions above. You may also include additional comments for the author, including concerns about dual publication, research ethics, or publication ethics. (Please upload your review as an attachment if it exceeds 20,000 characters)

Reviewer #2: Great work. All my comments are very well addressed. Reviewed the response given to comments by other reviewers and found they are also addressed well.

**********

7. PLOS authors have the option to publish the peer review history of their article (what does this mean?). If published, this will include your full peer review and any attached files.

If you choose “no”, your identity will remain anonymous but your review may still be made public.

**Do you want your identity to be public for this peer review?**  For information about this choice, including consent withdrawal, please see our Privacy Policy.

Reviewer #2: **Yes:**  Ganesh Babu Balu Subburaman

**********

[NOTE: If reviewer comments were submitted as an attachment file, they will be attached to this email and accessible via the submission site. Please log into your account, locate the manuscript record, and check for the action link "View Attachments". If this link does not appear, there are no attachment files.]

To ensure your figures meet our technical requirements, please review our figure guidelines: https://journals.plos.org/plosone/s/figures

You may also use PLOS’s free figure tool, NAAS, to help you prepare publication quality figures: https://journals.plos.org/plosone/s/figures#loc-tools-for-figure-preparation.

NAAS will assess whether your figures meet our technical requirements by comparing each figure against our figure specifications.

# Author comment: Author response to Decision Letter 2

**DOI:** 10.1371/journal.pone.0354744.r004

27 Jun 2026

Reviewer Comments

Thank you for the revised manuscript. The authors have attempted to incorporate a Health System Module into the existing RAAB framework, which is a welcome and potentially valuable initiative. However, there remain several concerns regarding the feasibility, interpretation, and practical utility of the proposed module and its findings.

Major Comments

1. Feasibility and Validation of the Health System Module

While integrating a Health System Module into RAAB is an interesting concept, it is unclear whether any pilot testing was conducted independently of the RAAB survey to assess the feasibility, completeness, and reliability of collecting such information.

Based on the data presented in the manuscript, the module appears to provide predominantly cataract surgery output-related information, while data related to diabetic retinopathy (DR) and refractive error services are minimal and incomplete. Therefore, statements such as: “It demonstrates how integrating robust epidemiological data with service capability profiling can reveal gaps that remain unseen when either type of data is considered separately” may be overstated and are not adequately supported by the current findings. The manuscript would benefit from a more balanced interpretation of what the Health System Module was actually able to capture in practice.

Our Response:

Thanks for your comment. As indicated in the title, this is the first implementation of this module, and we did not conduct a pilot. Although the module is intended to collect information on services for cataract, refractive error, and diabetic retinopathy, we agree with the reviewer that the available data were mostly limited to cataract delivery. The gaps in the available data were highlighted during implementation and are also noted in our conclusion section in the earlier submitted version - “In addition, the module can highlight gaps in the data required for regional planning.”

We have revised the statement referred to by the reviewer to read (lines 427-432):

“For cataract surgery volume, the analysis shows that integrating robust epidemiological data with service capability profiling can uncover gaps that remain hidden when either data type is analysed separately. However, due to a lack of data on cataract outcomes, refractive errors and DR, the same conclusion cannot be drawn for the assessment of the quality of cataract surgery delivery and the delivery of services for other eye health conditions.”

We have also reported the challenges in the implementation of the Health System Module in the discussion section now (Lines 446-451):

“Although the Health System Module provides the capacity to collect comprehensive data on the three eye conditions, our study highlights feasibility and operational limitations. Data completeness at the facility level, the feasibility of collecting data from small optical shops, reservations about sharing outcome data from private providers, and the lack of open-source outcome data are some of the challenges highlighted by our study. All these can reduce the programmatic utility of the Health System Module.”

We have also added the following in our conclusion(Line 458-460):

“Additionally, the module can identify gaps in the data required for regional planning. Its usefulness can be increased through improved data collection at the facility level and by improving the feasibility of data sharing.”

2. Recommendations Regarding a National Surgical Outcomes Portal

The authors recommend establishing a national portal where organizations could share cataract surgical outcomes anonymously. However, a similar initiative was attempted more than a decade ago by VISION 2020 India and was not successful.

This experience highlights that the challenge is not merely technological but also behavioural and systemic. While having such information would undoubtedly be valuable, successful implementation requires substantial behavioural change, institutional buy-in, trust, and sustained participation. Therefore, studies evaluating strategies to promote behavioural and system-level adoption may be more important at this stage than simply developing additional reporting modules that may prove difficult to implement consistently.

Our Response:

Thanks for this feedback. We have added this now in our discussion (lines 411-414):

“A few years back, a similar national-level initiative to capture cataract outcomes was attempted (27) but was unsuccessful. This highlights that the issue is not just technical but also behavioural and systemic. Research into strategies to encourage behavioural change and adoption is required.”

3. Programmatic Utility of the Health System Data

The manuscript does not clearly explain how the collected Health System Module data would directly inform program planning or improve service delivery in the surveyed region. The practical application of these data for district-level planning, referral strengthening, workforce deployment, or service improvement needs to be articulated more clearly.

I would therefore suggest that the authors substantially revise the sections related to the Health System Module, including a more detailed discussion of its feasibility, data completeness, operational limitations, challenges in implementation and potential programmatic utility

Our Response:

Thanks, we have added more details now

The following was included in the Introduction in the submitted version:

“This module collects information on policies related to eye services. It captures services offered by both government and non-government eye care providers for three common causes of vision loss: cataract, uncorrected refractive error, and diabetic retinopathy (DR). Since RAABs are typically conducted at the subnational level, this module provides a quick, user-friendly tool to support eye care planning within a survey area. “

We have added this in the introduction to highlight how it can support district-level planning (lines 78-85):

“While RAAB results can help estimate the burden of eye diseases, data on available services are also needed to plan at the district level. This module enables a relatively quick assessment of service gaps for cataract, refractive error, and DR services. The data to be collected in the module include the outputs and outcomes of cataract surgeries, the number of glasses dispensed, vision with glasses dispensed, the number of individuals screened and treated for DR and the human resources at the respective centres providing services. This can support the planning needed to scale up services, improve the service quality, and plan the workforce.”

And in the discussion section, we have highlighted the feasibility and challenges in implementation influencing the programmatic utility (Lines 446-451):

“Although the Health System Module provides the capacity to collect comprehensive data on the three eye conditions, our study highlights feasibility and operational limitations. Data completeness at the facility level, the feasibility of collecting data from small optical shops, reservations about sharing outcome data from private providers, and the lack of open-source outcome data are some of the challenges highlighted by our study. All these can reduce the programmatic utility of the Health System Module”

Additional Comments

4. Table 5

Table 5 provides very limited information and does not add substantial value to the manuscript in its current form. I would suggest removing the table and instead providing the Health System Module link/reference for interested readers.

Our Response:

Thanks for this feedback

We have removed Table 5 from the manuscript and added it as a supplementary table (S1_Table.word). We have renumbered the other supplementary tables accordingly. Instead of the table, we have added the following information in the Results section(Lines 270-279):

“A total of 14 service providers were found to be eligible, and all were included. These included 10 non-governmental organizations (NGOs), 2 government hospitals, and 2 private for-profit organizations. Permanent cataract services were provided by 10 providers, refraction services by 11, glasses dispensing by 9, and screening and treatment of DR by 2. Data on the number of cataract surgeries were available from 12 providers (86%), but only 1 facility (7%) had pre- and post-operative vision data. Data on the cost of cataract surgery for the patient were available from 9 providers (64%). Data on the number of refractions were available from 7 (50%) providers and on dispensed glasses from 4 providers (29%) that offer permanent or occasional refractive services. Data on DR screening or treatment were unavailable from all providers. Details on data availability are included in S1_Table.word.”

5. Interpretation of Corneal Blindness Findings

The manuscript states:

“Corneal opacities were the second leading cause of blindness…”

followed by a detailed description of the tele-ophthalmology-enabled cornea care system already in place.

However, if such systems are already functioning effectively, the persistently high burden of corneal blindness in the region requires further exploration. The discussion currently focuses primarily on available infrastructure rather considering, analysing and discussing the likely reasons for the high burden of corneal blindness in the region and propose targeted strategies to reduce it.

Our Response:

Thanks for this feedback. We agree that other interventions may be required. We have added that to the discussion (Lines 334-339):

“Although this improves the infrastructure for early intervention for corneal conditions among patients presenting to the health system, more needs to be done to promote prevention and encourage early presentations. In this predominantly rural region (7), agricultural trauma could be an important cause of corneal infections and blindness. (16) Targeted activities to prevent agricultural injuries, raise awareness of injury prevention, and emphasise the need to seek care early after an injury should be planned.”

6. Inconsistencies in Reported Numbers

There appear to be inconsistencies between the Results and Discussion sections.

For example, in the Discussion the authors state:

“The backlog of cataract surgeries for eyes with VA less than 6/12 … was approximately 130,000, with about 41,000 individuals needing surgeries in both eyes and roughly 48,000 in one eye…”

However, the numbers reported in the Results section differ. These discrepancies should be carefully reviewed and corrected to ensure consistency throughout the manuscript.

Our Response:

Thanks for this comment.

In the results section, we reported that an estimated 41,165 individuals had blindness or any degree of visual impairment (VA less than 6/12) due to cataract in both eyes. For individuals with poor vision in one eye due to cataract, we reported 41,984 for those with vision less than 6/18 ( not less than 6/12). The estimated number of individuals with vision in one eye less than 6/12 due to cataract was 47,840, and this number was included in Supplementary Table 2 in the original submission but not in the text (Supplementary Table 3 in the now-revised version). We have now included this figure in the text in results section. Thus, the backlog of cataract surgeries with a VA threshold of 6/12 is around 82,000 eyes across 41,000 individuals needing surgery in both eyes, plus an additional around 48,000 eyes belonging to individuals needing surgery in one eye. This totals approximately 130,000 eyes needing surgery at a threshold of 6/12, as mentioned in the discussion section.

The revised text in the result section is (Lines 230-235):

“Overall, out of the estimated 100,459 people with blindness or any degree of VI (including mild VI) (Table 1), 41,165 were attributed to unoperated cataract in both eyes (S3_Table.word). There were projected 23,829 individuals with MSVI or blindness due to cataract in both eyes. An additional 41,984 individuals with cataract in one eye with VA less than 6/18 and 47,840 individuals with VA less than 6/12 were estimated. (S3_Table.word)”

And in the discussion section (Line 358):

The backlog of cataract surgeries for eyes with VA less than 6/12 (the cut-off used by WHO for eCSC) was approximately 130,000, with about 41,000 individuals needing surgery in both eyes (82,000 eyes) and almost 48,000 in one eye ( S3_Table.word).

Overall Recommendation

The concept of integrating a Health System Module into RAAB is important and potentially valuable. However, the current manuscript overstates some conclusions relative to the available data and does not sufficiently address the feasibility, implementation challenges, behavioural aspects, and practical utility of the proposed approach.

Hence, substantial revision is required, particularly in relation to the interpretation and discussion of the Health System Module findings and their implications.

Our Response:

Thanks for this feedback. We agree with the reviewer that we did not highlight the feasibility challenges well.

We have now revised the manuscript as per the suggestions

Also, we have added to the discussion section that this module faces implementation and feasibility challenges. The fact that it addresses only three eye conditions was already reported as a limitation in the previous version. (“One limitation of the health system module is that it is intended to collect information only on cataract, refractive error, and DR”). We had also included the challenges in collecting data from all the optical shops in the previous version (“We also could not include information on refraction and glasses sold from optical shops due to logistical reasons.”)

Now we have added the following to the discussion section (Line 446-451):

“Although the Health System Module provides the capacity to collect comprehensive data on the three eye conditions, our study highlights feasibility and operational limitations. Data completeness at the facility level, the feasibility of collecting data from small optical shops, reservations about sharing outcome data from private providers, and the lack of open-source outcome data are some of the challenges highlighted by our study. All these can reduce the programmatic utility of the Health System Module.”

And the following to the conclusions (Line 458-460):

Additionally, the module can identify gaps in the data required for regional planning. Its usefulness can be increased through improved data collection at the facility level and by improving the feasibility of data sharing

[Reviewer's Responses to Questions]

Comments to the Author

1. If the authors have adequately addressed your comments raised in a previous round of review and you feel that this manuscript is now acceptable for publication, you may indicate that here to bypass the “Comments to the Author” section, enter your conflict of interest statement in the “Confidential to Editor” section, and submit your "Accept" recommendation.

Reviewer #2: All comments have been addressed

2. Is the manuscript technically sound, and do the data support the conclusions?

The manuscript must describe a technically sound piece of scientific research with data that supports the conclusions. Experiments must have been conducted rigorously, with appropriate controls, replication, and sample sizes. The conclusions must be drawn appropriately based on the data presented.

Reviewer #2: Yes

3. Has the statistical analysis been performed appropriately and rigorously?

Reviewer #2: Yes

4. Have the authors made all data underlying the findings in their manuscript fully available?

The PLOS Data policy requires authors to make all data underlying the findings described in their manuscript fully available without restriction, with rare exception (please refer to the Data Availability Statement in the manuscript PDF file). The data should be provided as part of the manuscript or its supporting information, or deposited to a public repository. For example, in addition to summary statistics, the data points behind means, medians and variance measures should be available. If there are restrictions on publicly sharing data—e.g. participant privacy or use of data f

Attachment Submitted filename: editor comments raab-hsm.docx

# Editor report: Decision Letter 2

**DOI:** 10.1371/journal.pone.0354744.r005

**Authors:** Rohit C. Khanna

8 Jul 2026

PONE-D-25-63896R2

Beyond Prevalence: A Complete Catchment Eye Health System Profile Using Rapid Assessment of Avoidable Blindness, a Population-Based Eye Health Survey,and the First Implementation of the Newly Developed Health System Module

PLOS One

Dear Dr. Sabherwal,

Thank you for submitting your manuscript to PLOS ONE. After careful consideration, we feel that it has merit but does not fully meet PLOS ONE’s publication criteria as it currently stands. Therefore, we invite you to submit a revised version of the manuscript that addresses the points raised during the review process.

Please submit your revised manuscript by Aug 22 2026 11:59PM. If you will need more time than this to complete your revisions, please reply to this message or contact the journal office at plosone@plos.org. When you're ready to submit your revision, log on to https://www.editorialmanager.com/pone/ and select the 'Submissions Needing Revision' folder to locate your manuscript file.

Please include the following items when submitting your revised manuscript:

- A letter that responds to each point raised by the academic editor and reviewer(s). You should upload this letter as a separate file labeled 'Response to Reviewers'.
- A marked-up copy of your manuscript that highlights changes made to the original version. You should upload this as a separate file labeled 'Revised Manuscript with Track Changes'.
- An unmarked version of your revised paper without tracked changes. You should upload this as a separate file labeled 'Manuscript'.

If you would like to make changes to your financial disclosure, please include your updated statement in your cover letter. Guidelines for resubmitting your figure files are available below the reviewer comments at the end of this letter.

If applicable, we recommend that you deposit your laboratory protocols in protocols.io to enhance the reproducibility of your results. Protocols.io assigns your protocol its own identifier (DOI) so that it can be cited independently in the future. For instructions see: https://journals.plos.org/plosone/s/submission-guidelines#loc-laboratory-protocols. Additionally, PLOS ONE offers an option for publishing peer-reviewed Lab Protocol articles, which describe protocols hosted on protocols.io. Read more information on sharing protocols at https://plos.org/protocols?utm_medium=editorial-email&utm_source=authorletters&utm_campaign=protocols.

As the corresponding author, your ORCID iD is verified in the submission system and will appear in the published article. PLOS supports the use of ORCID, and we encourage all coauthors to register for an ORCID iD and use it as well. Please encourage your coauthors to verify their ORCID iD within the submission system before final acceptance, as unverified ORCID iDs will not appear in the published article. *Only*  the individual author can complete the verification step; PLOS staff *cannot*  verify ORCID iDs on behalf of authors.

We look forward to receiving your revised manuscript.

Kind regards,

Rohit C. Khanna, MD, MPH

Academic Editor

PLOS One

Journal Requirements:

If the reviewer comments include a recommendation to cite specific previously published works, please review and evaluate these publications to determine whether they are relevant and should be cited. There is no requirement to cite these works unless the editor has indicated otherwise.

Please review your reference list to ensure that it is complete and correct. If you have cited papers that have been retracted, please include the rationale for doing so in the manuscript text, or remove these references and replace them with relevant current references. Any changes to the reference list should be mentioned in the rebuttal letter that accompanies your revised manuscript. If you need to cite a retracted article, indicate the article’s retracted status in the References list and also include a citation and full reference for the retraction notice.

Additional Editor Comments :

Based on the revised version, in abstract, modify the conclusion as "Integrating RAAB with a structured health system assessment enabled a unique, comprehensive situational analysis linking epidemiological needs with service capacity. It also highlighted data gaps, feasibility and operational limitations regarding data for cataract, refractive, and diabetic retinopathy services in the region. This model can guide regional planning and strengthen monitoring of effective coverage. However, challenges in data collection can reduce programmatic utility of the Health System Module"

[Note: HTML markup is below. Please do not edit.]

[NOTE: If reviewer comments were submitted as an attachment file, they will be attached to this email and accessible via the submission site. Please log into your account, locate the manuscript record, and check for the action link "View Attachments". If this link does not appear, there are no attachment files.]

To ensure your figures meet our technical requirements, please review our figure guidelines: https://journals.plos.org/plosone/s/figures

You may also use PLOS’s free figure tool, NAAS, to help you prepare publication quality figures: https://journals.plos.org/plosone/s/figures#loc-tools-for-figure-preparation.

NAAS will assess whether your figures meet our technical requirements by comparing each figure against our figure specifications.

# Author comment: Author response to Decision Letter 3

**DOI:** 10.1371/journal.pone.0354744.r006

9 Jul 2026

Additional Editor Comments :

Based on the revised version, in abstract, modify the conclusion as "Integrating RAAB with a structured health system assessment enabled a unique, comprehensive situational analysis linking epidemiological needs with service capacity. It also highlighted data gaps, feasibility and operational limitations regarding data for cataract, refractive, and diabetic retinopathy services in the region. This model can guide regional planning and strengthen monitoring of effective coverage. However, challenges in data collection can reduce programmatic utility of the Health System Module"

Response:

Thanks for your feedback. We have modified the conclusion in the abstract now to-

“Conclusion

Integrating RAAB with a structured health system assessment enabled a unique, comprehensive situational analysis linking epidemiological needs with service capacity. It also highlighted data gaps, feasibility and operational limitations regarding data for cataract, refractive, and diabetic retinopathy services in the region. This model can guide regional planning and strengthen monitoring of effective coverage. However, challenges in data collection can reduce the programmatic utility of the Health System Module.”

Attachment Submitted filename: Response to editors comments.docx

# Editor report: Decision Letter 3

**DOI:** 10.1371/journal.pone.0354744.r007

**Authors:** Rohit C. Khanna

12 Jul 2026

Beyond Prevalence: A Complete Catchment Eye Health System Profile Using Rapid Assessment of Avoidable Blindness, a Population-Based Eye Health Survey,and the First Implementation of the Newly Developed Health System Module

PONE-D-25-63896R3

Dear Dr. Sabherwal,

We’re pleased to inform you that your manuscript has been judged scientifically suitable for publication and will be formally accepted for publication once it meets all outstanding technical requirements.

Within one week, you’ll receive an e-mail detailing the required amendments. When these have been addressed, you’ll receive a formal acceptance letter and your manuscript will be scheduled for publication.

An invoice will be generated when your article is formally accepted. Please note, if your institution has a publishing partnership with PLOS and your article meets the relevant criteria, all or part of your publication costs will be covered. Please make sure your user information is up-to-date by logging into Editorial Manager at Editorial Manager® and clicking the ‘Update My Information' link at the top of the page. For questions related to billing, please contact billing support.

If your institution or institutions have a press office, please notify them about your upcoming paper to help maximize its impact. If they’ll be preparing press materials, please inform our press team as soon as possible -- no later than 48 hours after receiving the formal acceptance. Your manuscript will remain under strict press embargo until 2 pm Eastern Time on the date of publication. For more information, please contact onepress@plos.org.

Kind regards,

Rohit C. Khanna, MD, MPH

Academic Editor

PLOS One

Additional Editor Comments (optional):

Nil

Reviewers' comments:

# Editor report: Acceptance letter

**DOI:** 10.1371/journal.pone.0354744.r008

**Authors:** Rohit C. Khanna

PONE-D-25-63896R3

PLOS One

Dear Dr. Sabherwal,

I'm pleased to inform you that your manuscript has been deemed suitable for publication in PLOS One. Congratulations! Your manuscript is now being handed over to our production team.

At this stage, our production department will prepare your paper for publication. This includes ensuring the following:

* All references, tables, and figures are properly cited

* All relevant supporting information is included in the manuscript submission,

* There are no issues that prevent the paper from being properly typeset

You will receive further instructions from the production team, including instructions on how to review your proof when it is ready. Please keep in mind that we are working through a large volume of accepted articles, so please give us a few days to review your paper and let you know the next and final steps.

Lastly, if your institution or institutions have a press office, please let them know about your upcoming paper now to help maximize its impact. If they'll be preparing press materials, please inform our press team within the next 48 hours. Your manuscript will remain under strict press embargo until 2 pm Eastern Time on the date of publication. For more information, please contact onepress@plos.org.

You will receive an invoice from PLOS for your publication fee after your manuscript has reached the completed accept phase. If you receive an email requesting payment before acceptance or for any other service, this may be a phishing scheme. Learn how to identify phishing emails and protect your accounts at https://explore.plos.org/phishing.

If we can help with anything else, please email us at customercare@plos.org.

Thank you for submitting your work to PLOS ONE and supporting open access.

Kind regards,

PLOS ONE Editorial Office Staff

on behalf of

Dr. Rohit C. Khanna

Academic Editor

PLOS One
